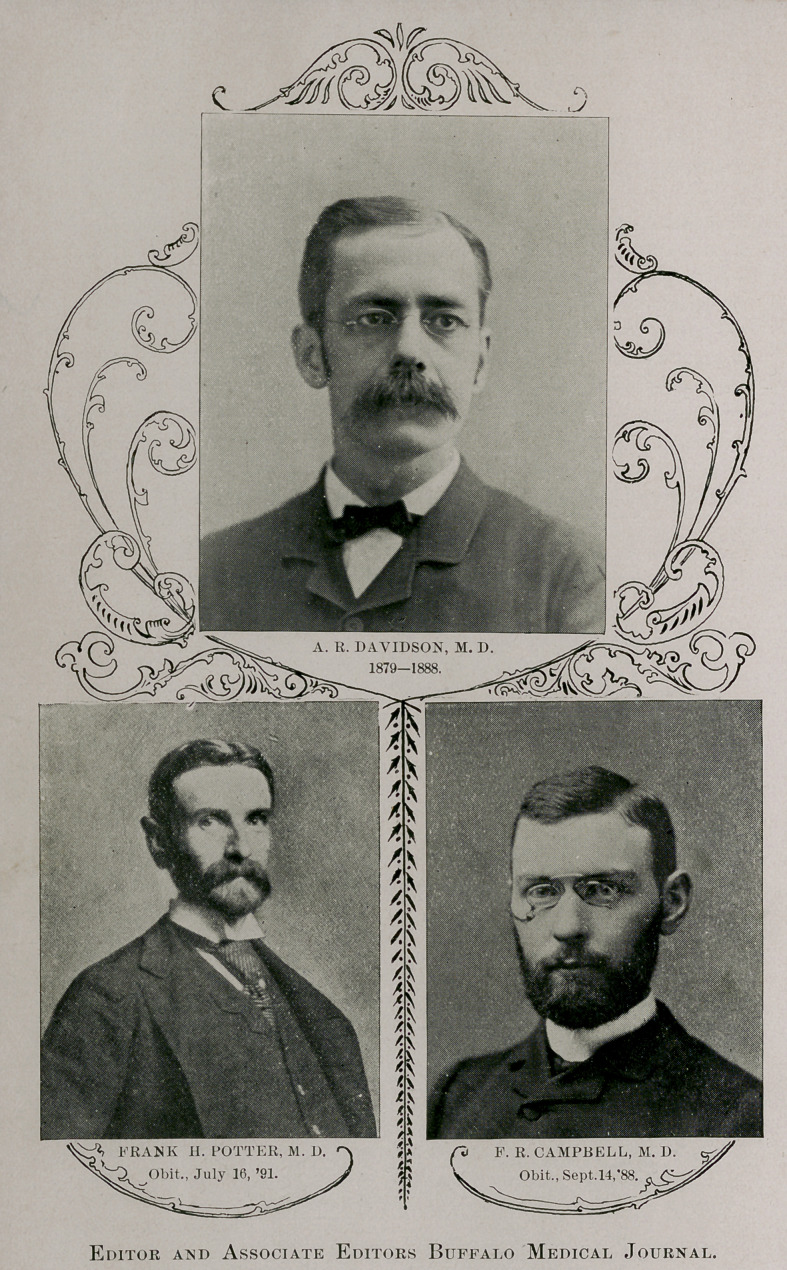# Fifty Years of Medical Journalism

**Published:** 1895-08

**Authors:** William Warren Potter


### Special Article.

### 1845—THEN AND NOW—1895.

*Fifty Years of Medical Journalism in Buffalo—A Historical Reminiscence—Medical Journals—Medical Colleges—Hospitals—Medical Societies.*

### I.—The Buffalo Medical Journal.

### Epoch I.—1845–1855.

IN JUNE, 1845, the first number of the Buffalo Medical Journal was published, under the editorship of Dr. Austin Flint, who was also its founder and owner. It was printed by Jewett, Thomas & Co., at the office of the *Commercial Advertiser*, and consisted of twenty unleaded standard octavo pages, in long primer type. It contained an introductory by the editor that occupied two pages and a quarter; notes of a European tour, by Dr. F. H. Hamilton, professor of surgery in Geneva medical college; cases of acute rheumatism treated with nitrate of potash in large doses, by Dr. Alden S. Sprague; case of aortitis, with autopsy and remarks, by Dr. George N. Burwell; case of hydrophobia, reported by Dr. James P. White, and cases of midwifery with twins at different stages of development, by Dr. H. N. Loomis. The last four pages of this number were filled with paragraphs under the general head—*editorial, medical intelligence, bibliographical notices*, etc.

This was the first medical journalistic venture between New York and Cincinnati or St. Louis, in the southwest, and naturally arrested the attention of the medical fraternity between the Atlantic and the Mississippi. Its establishment was the result of much thought on the part of the editor, many conferences with his professional colleagues and not a little perturbation of spirit among those interested in its success.

At that time Buffalo contained less than 30,000 inhabitants, and though there were about seventy physicians of all sorts and conditions, one half of which were regulars, there was yet no organized medical society nor other cohesive force or center round which physicians might rally. Nevertheless, the journal was a success from the start, as might be expected from the energy and character of the man who had seated himself on its editorial tripod, as well as those who were his immediate advisers. The first volume contained only 284 pages; but the second volume grew to the aggregate of 758 pages, so great was the demand for space.

BUFFALO

MEDICAL JOURNAL

AND

MONTHLY REVIEW

OF

MEDICAL AND SURGICAL SCIENCE.

EDITED BY AUSTIN FLINT, M. D.

**Vol. I.—JUNE.—No. I.**

BUFFALO:

PUBLISHED BY JEWETT, THOMAS & CO.

Office of Commercial Advertiser.

1845.

FACSIMILE OF FIRST COVER-PAGE OF FIRST NUMBER OF BUFFALO MEDICAL JOURNAL, ONE-THIRD SIZE.

Mr. James N. Matthews, afterward editor and proprietor of the *Buffalo Morning Express*, and the head of the famous art printing house of Matthews, Northrup & Co., worked as a compositor on the first numbers of the JOURNAL, and he informed the writer, in a conversation held a few months before his death, that at first he experienced great difficulty in deciphering Dr. Flint's "copy."

The history of the BUFFALO MEDICAL JOURNAL involves the history of the medical profession in Buffalo for the past fifty years. In its pages are recorded the principal medical events that

JAMES N. MATTHEWS,

First compositor on the BUFFALO MEDICAL JOURNAL,

Editor and Owner *Buffalo Morning Express*.

have occurred here during the half century of its existence, some of which have been given with considerable detail. It contains the reports of clinical cases that served to make the men of that period famous. In its fourth number is published the first information concerning the true nature of the infection of typhoid fever. A well at North Boston, Erie county, became poisoned by

VIEW OF BUFFALO, 1845.

the excreta of a typhoid patient brought from Massachusetts. Twenty-one cases occurred in five families, all living within a few rods of the fatal well and deriving their water supply from that source, of whom seven died. Dr. Austin Flint visited the locality, diagnosticated and traced an infectious disease, then unknown in this region, from New England to the hamlet of North Boston, dis-

JAMES PLATT WHITE, M. D.

tinctly established its contagion and pointed out its source. Nothing was omitted that could contribute to the completeness and convincing power of the evidence adduced ; the published report has become a classic in medical literature, and it formed the basis of a series of essays published in this journal by the distinguished investigator. This was Dr. Flint's first conspicuous success, and it is more than probable that it laid the foundation for his future fame as a clinician.

Dr. Frank Hastings Hamilton published in the pages of this journal his surgical clinics and fracture tables, from which sprang thematerial to construct his treatise on fractures and dislocations, a work that stands unrivaled as authority on the subject; it is a recognised classic in every country and in all tongues where medicine is known as a science.

Dr. James P. White lent his powerful influence in support of the JOURNAL from the first, and published in its columns essays and clinical reports, on which was laid the basis of a medical career that so justly made him famous in two hemispheres.

FRANK HASTINGS HAMILTON, M. D.

Dr. Flint conducted the JOURNAL as sole editor from 1845 until 1853,—eight years,—when, having been invited to teach the practice of medicine at Louisville, Ky., it became necessary to transfer it to other hands. Meanwhile, a young man from the country had been contributing a series of articles to its columns under the name of "Smelfungus," that had attracted great attention, on account of their rare wit, wisdom and originality. Circumstances arose, which will be alluded to hereafter, that made it convenient for this young physician to transfer his residence to Buffalo. Recognising his talent and fitness for the work, Dr. Flint made haste to invite "Smelfungus" to become associated with him in the editorial conduct of the JOURNAL. Thus, Dr. Sanford B. Hunt,—"Smelfungus" no longer,—without experience in journalism—indeed, almost without experience of any kind—by force of fortuitous circumstances, became practically the editor-in-chief with the issue for July, 1853. The wisdom of this choice on the part of Dr. Flint was never challenged, and two years later he conveyed his entire interests in the JOURNAL to Dr. Hunt; so, in June, 1855, the latter became sole editor and proprietor. Dr. Flint had, meanwhile, transferred his labors to other fields, after having been for a decade closely identified with the interests of the magazine, most of the time as sole editor and owner. With his departure closes what may be termed the first epoch of the JOURNAL.

### EPOCH II.—1855–1860.

During Dr. Hunt's administration, from 1853 to 1858, it may be said that the JOURNAL enjoyed the most brilliant period in its career. Putting his whole talent and energy into the work, the editor soon made his journal famous, not less for the sparkling originality of its editorial department, than for its journalistic *esprit de corps*. Dr. Hunt was a ready writer, an original thinker and a companionable man. Indeed, he was a genius with special aptitude for editorial work. His ideas ran so much faster than his pen that it was difficult for him to keep his thoughts in check while his pen caught up to his expressions. What a blessing to him the present fashion of stenographers would have been.

It was during this epoch that the journal had its first experience as defendant in a libel suit, an experience that has been repeated once since, yet with less material benefit to the plaintiff. The circumstances leading to the first one may be thus briefly summarised: Dr. John D. Hill had been expelled from the medical society of the county of Erie for a violation of its rules, and the JOURNAL had seen fit to make fearless and independent comment thereon. Fancying himself injured thereby, he brought suit for libel against the editors, Drs. Flint and Hunt. The result was announced in the issue for March, 1856, as follows: "In the libel suit brought by Dr. John D. Hill vs. Austin Flint and Sanford B. Hunt, as editors of the BUFFALO MEDICAL JOURNAL, which has come to trial since our last issue, the jury—(and *such* a jury!)—brought in a verdict of damages to the plaintiff to the amount of $500." Dr. Hill was subsequently restored to membership in the society and in 1887 was chosen its president.

In addition to his duties as editor, Dr. Hunt was professor of anatomy in Buffalo medical college and city editor of the *Commercial Advertiser*. Eventually he became anchored at the editorial desk of this daily newspaper, and surrendered the professorship above alluded to. A little later he was elected superintendent of public schools in Buffalo, soon after which the war came. He enthusiastically joined the army as surgeon of U. S. volunteers, and in 1863 was placed in charge of convalescent camp near Alexandria, Va. After the war he edited the volumes known as the history of the U. S. sanitary commission, and upon completion of this task he became editor of the Newark, N. J., *Daily Advertiser*. Finally he purchased the Sunday edition, known as the *Sunday Call*, now conducted by his son. In January, 1884, he was seized with a fatal illness, of which he died at Irvington, a suburb of Newark, April 26, 1884. His ashes repose in Forest Lawn, at Buffalo. The writer was his pupil in 1854–55, and feels it but just to make the foregoing record somewhat detailed in regard to his illustrious preceptor.

From 1858 to 1860, Dr. Austin Flint, Jr., was the editor and proprietor. Now came a period of disaster. This arose from divorcing the editorial and publishing departments. The prosperity that had hitherto attended the JOURNAL seemingly was at an end. Mr. A. I. Mathews, the then well-known druggist, obtained its ownership and soon prostituted its advertising columns to the printing of quack advertisements. Thereupon the profession withdrew its support and as a consequence the JOURNAL ceased publication. The fifteenth volume closed with the issue for May, 1860, and with it the second epoch came to an end.

### EPOCH III.—1861–1879.

The circumstances that led to the suspension of the publication of the JOURNAL need not be discussed in detail. Suffice it to say that Dr. Austin Flint, Jr., who resisted the action of the druggist before alluded to, with all his might, had moved to New York, and that the mismanagement of the publisher, Mr. Mathews,[1] led to a loss of confidence of the professional public in its usefulness and integrity. Without this confidence no medical journal can exist. Plans soon began to be discussed among leading medical men looking to its resuscitation, but these were always embarrassed by the fact that Mr. Mathews owned a proprietorship in the name and fame of the defunct journal.

Finally, however, these difficulties were overcome, and in

1. This Mathews must not be confounded with Mr. James N. Matthews, whose picture appears on page 67.

August, 1861, the journal was revived under the able editorship of Dr. Julius F. Miner, the well-known surgeon. A slight modification in name, however, became necessary, so it was called the BUFFALO MEDICAL AND SURGICAL JOURNAL AND REPORTER. It required no little courage and energy on the part of its editor to launch a medical journal at the time mentioned. The country had just plunged into civil war, and there was as a consequence deep commercial depression and distress, a period unfavorable for the commencement and growth of any enterprise requiring outlay of thought and expenditure of money. But the physicians of Buffalo had learned to appreciate the value of a good medical journal, all the more, perhaps, since they had been deprived of their own. So, nothing daunted, the indefatigable editor issued the first number of the new journal, which was in reality only a reëstablishing of the old one. The first number contained thirty-two pages, and the first volume an aggregate of 380 pages. With the beginning of the second volume of this new series the words "and Reporter" were dropped from its title, and it has been published since that time under the name of the BUFFALO MEDICAL AND SURGICAL JOURNAL. With a view, however, to simplicity, it will now resume its former name and will be known hereafter as the BUFFALO MEDICAL JOURNAL. For eighteen years Dr. Miner continued to edit and publish the JOURNAL, but he was assisted a portion of the time by Dr. Edward N. Brush, as associate editor. Dr. Brush is now superintendent of the Sheppard Asylum for the Insane in Baltimore county, Md.

During the period of the war the pages of the JOURNAL became a historical record of the medical officers who entered the military service from Buffalo and vicinity. In June, 1869, is found a record of the first application of the principle of enucleation to ovarian and other abdominal tumors as performed by its originator, the editor, Dr. Julius F. Miner.

### EPOCH IV.—1879–1895.

In 1879, Dr. Miner's failing health warned him to give up a portion of his work, the necessity for which had been a long time foreseen by his friends. Consequently the JOURNAL was sold to a syndicate, composed of Drs. Thomas Lothrop, A. R. Davidson, Herman Mynter, Lucien Howe and P. W. Van Peyma, whose administration began with volume XIX., new series, August, 1879. The first volume published under the new management contained 556 pages printed with small pica type— a larger size than that used in the preceding issues. With volume XXII., beginning August, 1882, the names of Drs. Howe and Mynter disappeared from the editorial staff, and two years later Dr. Van Peyma retired, leaving the JOURNAL in the hands of Drs. Lothrop and Davidson, the latter continuing as managing editor until his death, May 25, 1888.

In July, 1888, Dr. Davidson's interest in the magazine as well as his functions as managing editor passed to the hands of Dr. William Warren Potter, who has continued in their exercise since that time. The three editors during the first series are dead; so too are Dr. A. R. Davidson, former managing editor, and Drs. F. R.

Campbell and Frank Hamilton Potter, associate editors, whose obituaries have heretofore appeared in these columns. Thus, since the establishment of the JOURNAL, fifty years ago, six deaths have occurred in its editorial ranks. We present in this issue pictures of these our illustrious deceased predecessors and collaborators.

It would be manifestly improper in this article to speak in detail of the JOURNAL'S record during this epoch. It is too recent

THOMAS F. ROCHESTER, M. D.

and too well known to discourse upon now with due regard for delicate propriety. The editors chiefly responsible for its course are still at work striving to make it deserving of continued respect and confidence. Perhaps the historian of the future may have something to say of the fourth epoch in the JOURNAL'S career.

During the lifetime of the JOURNAL nearly all the improvements in medicine and surgery that are valuable have been developed, for it must be remembered that it has witnessed the introduction of anesthesia; the perfection of the stethoscope; the practical use of the speculum, the laryngoscope, the otoscope, the ophthalmoscope and the endoscope ; the use of the clinical thermometer ; the invention of the sphygmograph, the hypodermic syringe, the aspirator and the Esmarch bandage; the revelations of the microscope; asepsis and antisepsis as applied to surgery and obstetrics ; the rise and progress of the science of bacteriology ; the development and perfection of abdominal surgery as related to the removal of new growths ; the repair of the intestines for traumatism; the surgical treatment of appendicitis ; the surgery of the gall-bladder ; the rise and development of gynecological surgery; improved methods in treatment of fractures—all these the JOURNAL has witnessed and recorded in its pages, besides many others of great importance which neither time nor space permit us to mention. Whoever, therefore, has been wise enough to preserve and bind his journals from the outset has a valuable record of progress in medical science.

Though a number of journals have appeared in Buffalo during the lifetime of this one, they have, speaking generally, been short-lived and are now extinct. At the present time it is the only medical journal published in the area bounded on the north by Toronto, on the east by Rochester, on the south by Pittsburg and west by Cleveland and Detroit. The *Buffalo Dental Advertiser*, a quarterly, is a magazine that the JOURNAL is proud to acknowledge as a neighbor.

As a testimonial to the valued support that the JOURNAL has received from its contributors, subscribers and advertisers it now offers itself in an enlarged form and a new dress, for it well understands that it cannot hope to succeed without the continued favor of the medical profession, which it has enjoyed so long. It proposes to do all it can to deserve a continuance of professional approbation. It will continue in the future, as in the past, to attempt to reflect the opinions of the whole profession of medicine in this region on scientific questions and on the progress of medicine, and it proposes to labor anew to maintain the unification of the profession and for the advancement of medical science. It is especially devoted to the principles of higher medical education, and believes that these principles are best exemplified in the maintenance of state medical examining and licensing boards. It will advocate the establishment of such boards in those states that have not yet adopted the plan, as well as a higher standard of preliminary education.

This is the fiftieth year of its publication. While it is old in years, it must be young in activity, and it proposes to indicate its youthfulness by donning new garments, manifesting new energy, increasing the number of its pages, and otherwise improving itself so as to make it worthy to stand in the front rank with the best

GENERAL ALBERT J. MYER.

medical journals of the land. In greater Buffalo there will be a greater BUFFALO MEDICAL JOURNAL.

---

### II.—MEDICAL COLLEGES.

### MEDICAL DEPARTMENT, UNIVERSITY OF BUFFALO.

### EPOCH I.—1846–1850.

In 1846, authority was granted by the legislature of New York permitting the establishment of a medical school at Buffalo under a university charter. The subject had been agitated for several years, but formal steps preparatory to the application were not taken until the autumn of 1845. Public announcement of the success of the enterprise was made in the BUFFALO MEDICAL JOURNAL, September, 1846, stating that the medical department had been fully organized by creating seven professorships, to which the council of the University had made the following appointments: Chemistry and pharmacy, James Hadley, M. D.; physiology and medical jurisprudence, Charles B. Coventry, M. D.; general and special

FIRST BUFFALO MEDICAL COLLEGE, WASHINGTON AND SENECA STS.

anatomy, James Webster, M. D.; pathology and materia medica, Charles Alfred Lee, M. D.; principles and practice of surgery and clinical surgery, Frank Hastings Hamilton, M. D.; obstetrics and diseases of women and children, James Platt White, M. D.; principles and practice of medicine and clinical medicine, Austin Flint, M. D. Five of the seven chairs above named were filled by incumbents of professorships in Geneva Medical College which soon afterward was discontinued. Dr. Hamilton removed to Buffalo, Dr. Webster retained his residence in Rochester, Dr. Coventry continued to reside in Utica and Dr. Hadley's son, George, delivered the chemistry lectures from the first. One other name deserves mention in this connection,—that of Corydon L. Ford, M. D.,—who was appointed demonstrator of anatomy and who afterward became one of the most famous anatomists of modern times. Drs. White and Flint were the chief promoters of this college enterprise, though they were ably seconded by Mr. O. H. Marshall and other gentlemen not physicians. Hon. N. K. Hall,

THE SECOND BUFFALO MEDICAL COLLEGE—MAIN AND VIRGINIA STREETS.

afterward postmaster-general, was the representative in the assembly whose chief efforts obtained the charter. Millard Fillmore, then president of the United States, was the first chancellor of the University, an office which he continued to fill until his death, March 8, 1874. His portrait, given on p. 73, is from a steel engraving taken about the time he was president. Thus began the first permanently successful effort to establish in Buffalo an educational institution above the grade of common schools.

The first course of medical lectures opened February 24, 1847, with an attendance of sixty-six registered students, one of whom was Mr. L. G. Sellstedt, the distinguished artist, of this city, who was taking an optional course. The council of the university leased for a term of years the First Baptist church, that then stood on the corner of Seneca and Washington streets, the site of the present post office building. The BUFFALO MEDICAL JOURNAL announced, in its issue for December, 1846:

The council has been fortunate in obtaining a building admirably adapted for a medical college. Had it been erected with a view to this purpose it could hardly have been improved. A pictorial representation of the college accompanies the annual circular which has lately been issued.[1]

This building was used by the college during its first three academic years.

### EPOCH II.—1850-1893.

In 1849, the construction of the building on the corner of Main and Virginia streets was begun. It was completed in season for the fourth lecture course, 1849-50, at a cost of about $15,000. Public-spirited citizens gave the building and made no reckoning, among whom were A. D. Patchen, whose name heads the list with a subscription for $500; next came Jesse Ketchum, who gave $600, the largest single donor, and then followed in their order, A. H. Tracy, $200; George W. Tifft, $200; E. G. Spalding, $200 and Jabez Goodell, $200. There were eighty citizens who subscribed $100 each, and the remainder was raised in sums of $40 and $60, until the aggregate reached $12,000. The state gave $2,000 which made a sufficient amount to warrant the commencement of the construction of the new edifice. It was during this fourth year that Dr. White introduced demonstrative or clinical midwifery, a method of teaching that had already been established in Europe, but had never been attempted before in this country. A woman two weeks before confinement entered the janitor's apartments, where she boarded, and was cared for by the janitor's wife. After labor began the graduating class, 22 in number, assembled in an adjoining room and one by one, under the supervision of Professor White, they were admitted to the confinement room and were permitted to make vaginal examinations during the progress of labor. On the termination of the second stage all were assembled in the lying-in room and per-

1. We have succeeded in finding an old cover page of this circular, kindly loaned by Mrs. S. J. Reid, Prospect avenue, Buffalo, from which an exact copy is given on page 78.

mitted to witness the passage of the head over the perineum and the method employed to support the latter. This was all: there was no undue exposure of the woman and she made rapid convalescence, yet seldom has an event occurred that so completely shook the foundations of society in any city as did this. The newspapers commented upon it, doctors denounced it as "immoral" and a suit for libel followed. A scathing critique signed "L" appeared in one of the daily newspapers reflecting so intemperately

MILTON GROSVENOR POTTER, M. D.

upon Dr. White's course, that he promptly brought suit for libel against Dr. Horatio N. Loomis, the supposed author of the article. A trial ensued lasting four days, able counsel appeared on both sides, two stenographers were employed by the complainant (this was before the days of court stenographers) and a full report was made and published to the world. Much stress had been laid by the counsel for the defendant upon the fact that "public opinion" placed the stamp of its emphatic disapproval upon the course of Dr. White. Mr. Justice Mullett, who presided at the trial, swept all such fallacies from the jury box in a terse and eloquent charge, from which we quote as follows :

Public opinion has never been deemed a very safe agent in the administration of justice since it profaned the judgment seat and insulted heaven by the cry of Crucify Him ! Crucify Him ! ! Pilate, weak and timeserving, disobeyed the dictates of his own conscience and followed the popular outcry, which he mistook for public opinion. But the sacred history of that awful tragedy informs us that the chief priests and elders persuaded the multitude.

Dr. Loomis was acquitted, for it was proven that another had written the libel, but Dr. White was vindicated and his name will roll down the centuries in connection with the clinical teaching of midwifery in the United States, as being the first teacher to attempt it in this country.

In 1851, Dr. John C. Dalton, who had been appointed professor of physiology to succeed Dr. Coventry, and who was himself the pupil of the great Bernard, introduced here, for the first time in any medical college in this country, the plan of illustrating his lectures by vivisections before the class. Dr. Austin Flint, editor of this journal, taught the practice of medicine from 1846 to 1853, when he was succeeded by Dr. Thomas F. Rochester, who taught in this department until his death, May 24, 1887. Dr. Sanford B. Hunt was appointed demonstrator of anatomy in 1853, succeeded to the professorship in 1854, holding until 1858, when Dr. Sanford Eastman, an alumnus of the college, was appointed. On the death of the latter Dr. Milton G. Potter, also an alumnus, became professor of anatomy and served as such until his demise, January 28, 1878.

After Dr. Hamilton removed to New York in 1860, Dr. E. M. Moore, of Rochester, N. Y., who had been teaching surgical pathology, was appointed to the chair of surgery. Professor Moore continued his labors in this field until 1883. He is still in good health, though less actively at work than formerly.

Since it is my purpose in this article to speak especially of first things, I must be brief, passing many points of importance that it would be interesting to recall. In 1871, the subject of organizing an alumni association was agitated, and many conferences were held between members of the faculty and prominent alumni. An organization was finally perfected with Dr. Thomas D. Strong, of Westfield, as the first president. The first public meeting was held February 23, 1875, when the address to the alumni was delivered

THE PRESENT (THIRD) BUFFALO MEDICAL COLLEGE, HIGH STREET—MEDICAL DEPARTMENT, UNIVERSITY OF BUFFALO.

by Dr. William Warren Potter in St. James's Hall (that stood on the site of the Iroquois Hotel) on the evening of commencement day. At the first banquet of the association, held at the Tifft House, on the same evening, Dr. T. D. Strong presided, grace was said by the Rev. G. W. Heacock, D. D., and Professor James P. White responded to the first toast, "Our Alma Mater."

While it would be interesting in this connection to mention the achievements of some of the distinguished alumni, there is now neither time nor space to do so. Yet there is one whose name and fame have become coëxtensive with the boundaries of the globe itself. Albert J. Myer graduated in the class of '51, entered the United States Army as assistant surgeon in 1854. and soon afterward was assigned to duty in Texas. While there he devised a mono-manual deaf-mute alphabet. Still later he invented and put into practical operation a system of military signals, that contributed amazingly to the success of our arms in the late unhappy war. A separate bureau was created by act of congress and Dr. Myer was placed at its head with the rank of brigadier-general. General Myer, gifted with a scientific mind associated with inventive genius, prepared a code of weather signals that has become the basis of the present system in operation throughout the world, which gained for him the familiar title of "Old Probabilities."

He died at Buffalo, August 24, 1880, and his remains rest in a beautiful mausoleum in Forest Lawn cemetery.

### Epoch III.

A few years ago the college building at the corner of Main and Virginia streets became unsuited to the purposes of modern medical teaching. Its anatomical rooms were inadequate, its laboratories too restricted in area and, in short, the methods of 1890 had outgrown those of 1850. It must not be forgotten, however, that this was the first building erected in this city for collegiate instruction since the soil on which it stands was relinquished by the Senecas. It ought, therefore, in our view, to be preserved by the city as a memorial to educational advancement. It might be made a school library building or a museum of some sort, that would perpetuate the memory of the men who founded it as well as the purposes for which it was founded.

The present college building was opened March 5, 1893, with public ceremonies, an account of which appeared in the Journal,

April, 1893. It is a superb building, admirably adapted to the purposes of medical instruction, and bespeaks the energy and sagacity of its projectors. We need not extend our comments in detail during this epoch. It pertains to the immediate present, and its history is as yet unwritten. Beginning with the mention of the first faculty of seven, it is fitting that we should name the present one that is conducting the school on such prosperous lines. The successors, then, of the original seven are :

Charles Cary, M. D., professor of materia medica, therapeutics and clinical medicine ; Matthew D. Mann, A. M., M. D., dean, professor of obstetrics and gynecology ; Roswell Park, A. M., M. D., professor of principles and practice of surgery and clinical surgery ; Julius Pohlman, M. D., professor of physiology ; Charles G. Stockton, M. D., professor of principles and practice of medicine and clinical medicine ; John Parmenter, M. D., secretary, professor of anatomy and adjunct professor of clinical surgery ; Herbert M. Hill, A. M., Ph.D., professor of chemistry, toxicology and physics.

To these are now added seven adjunct professors, as follows : Wm. C. Phelps, M. D., associate professor and demonstrator of anatomy ; De Lancey Rochester, A. M., M. D., adjunct professor of the principles and practice of medicine ; P. W. Van Peyma, M. D., adjunct professor of obstetrics ; Eli H. Long, M. D., adjunct professor of materia medica ; Herbert U. Williams, M. D., professor of pathology ; Fred. B. Willard, M. D., assistant demonstrator of anatomy ; Loren H. Staples, M. D., prosector to the chair of anatomy.

The professors of special departments are : Lucien Howe, A. M., M. D., clinical professor of ophthalmology ; Mahlon B. Folwell, M. D., clinical professor of diseases of children ; Ansley Wilcox, A. B., LL. B., professor of medical jurisprudence ; D. W. Harrington, M. D., professor of venereal diseases ; Henry R. Hopkins, M. D., professor of hygiene ; Bernard Bartow, M. D., clinical professor of orthopedic surgery ; F. Whitehill Hinkel, M. D., clinical professor of laryngology ; Ernest Wende, B. S., M. D., clinical professor of dermatology ; W. E. Ford, A. M., M. D., Utica, N. Y., professor of electro-therapeutics ; Jas. W. Putnam, M. D., clinical professor of diseases of the nervous system ; Wm. H. Heath, M. D., clinical professor of genito-urinary and venereal diseases ; William C. Barrett, M. D., D. D. S., professor of oral pathology ; Floyd S. Crego, M. D., professor of insanity and diseases of the brain ; Willis G. Gregory, M. D., Ph. G., director of the pharmacal laboratory ; Francis T. Metcalfe, M. D., lecturer on comparative pathology ; Franklin W. Barrows, A. B., M. D., lecturer on histology and biology ; F. J. Thornbury, M. D., lecturer on bacteriology.

The instructors are: Fred B. Willard, M. D., instructor in anatomy ; Allen A. Jones, M. D., instructor in medicine ; A. L. Benedict, A. M., M. D., instructor in materia medica and therapeutics ; M. A. Crockett, A. B., M. D., instructor in obstetrics and gynecology; Edward J. Meyer, M. D., instructor in surgery; Albert T. Lytle, M. D., instructor in chemistry ; Dewitt H. Sherman, M. D., instructor in therapeutics; Ferdinand G. Moehlau, M. D., instructor in physiology ; J. F. Whitwell, M. D., instructor in general pathology ; H. C. Rooth, M. D., instructor in special pathology ; E. L. Frost, M. D., instructor in obstetrics.

Student assistants : F. C. Busch, B. S., A. T. Kerr, B. S., assistants in pathology and in the histological laboratory.

Clinical instructors : General practice, Drs. Allen A. Jones, Geo. Himmelsbach, A. T. Lytle ; surgery, Drs. Edward J. Meyer, J. Franklin Whitwell ; diseases of women, Drs. M. A. Crockett, P. C. Cornell ; diseases of the nervous system, Drs. James W. Putnam, F. S. Crego, James A. Gibson ; diseases of children, Drs. Irving M. Snow, Maude J. Frye ; diseases of the skin, Drs. Ernest Wende, Grover Wende ; diseases of the nose and throat, Drs. Henry J. Mulford, Geo. F. Cott ; diseases of the eye and ear, Drs. Julius Pohlman, Elmer Starr, H. Y. Grant ; diseases of the genito-urinary system, Drs. Loren H. Staples, F. G. Moehlau ; obstetrics, Drs. W. G. Bissell, Chas. A. Clements, Irving W. Potter, H. Mead, E. L. Frost.

This array of teachers, contrasted with the original seven, indicates the progress of medical instruction during the last fifty years. As a further evidence of progress it may be mentioned that the following departments have been erected in the University of Buffalo since the establishment of the medical department, namely—the department of pharmacy, established 1886 ; the department of law, established 1887 ; the department of dentistry, established 1892, and the school of pedagogy, established in 1895.

### II.—MEDICAL DEPARTMENT, NIAGARA UNIVERSITY.

From 1847 to 1883, Buffalo University medical college occupied alone the field of medical training in this city. In the latter year, however, prompted by the desire to advance the standard of medical education, a number of physicians petitioned the regents of the University of the State of New York for authority to establish another medical college in Buffalo. In 1863, an academic school called the Seminary of Our Lady of Angels was located near Suspension Bridge. In 1883, this seminary was erected into a university with authority to locate any of its colleges in Erie county. A department of medicine was thereupon organized by Niagara University, which was located in the City of Buffalo. Its requirements for admission were that students must pass a matric-

CHARLES C. F. GAY, M. D.

ulation examination in such branches as were considered necessary to fit them for the study of medicine. It was announced that the course of study would continue during three years, to comprise three full courses of lectures of six months each and the faculty recommended that students should extend their studies to four years. The first faculty was organized as follows: John Cronyn, M. D., professor of the principles and practice of medicine and clinical medicine, president of the faculty; Thomas Lothrop, M. D., professor of obstetrics; William H. Heath, M. D., professor of descriptive and surgical anatomy; Augustus R. Davidson, M. D., professor of medical chemistry, pharmacy and toxicology; Henry D. Ingraham, M. D., professor of gynecology and diseases of children; Charles G. Stockton, M. D., professor of materia medica and therapeutics; Charles C. F. Gay, M. D., professor of operative and clinical surgery; William S. Tremaine, M. D., professor of the principles and practice of surgery and clinical surgery; Clayton M. Daniels, M. D., professor of clinical surgery and adjunct professor of surgery; George E. Fell, M. D., professor of physiology and microscopy; Alvin A. Hubbell, M. D., professor of ophthalmology, otology and laryngology; Hon. Joseph M. Congdon, professor of jurisprudence. The right reverend Stephen V. Ryan, D. D., was announced as chancellor of the University and John L. C. Cronyn, M. D., as demonstrator of anatomy. Of these Drs. John Cronyn, Lothrop, Ingraham and Hubbell still remain in their original places; Drs. Davidson and Gay are dead; Drs. Stockton and Heath are teaching in Buffalo University and Drs. Tremaine, Daniels, Fell, John L. C. Cronyn and Hon. Joseph M. Congdon have resigned. Dr. Gay was distinguished as a surgeon, having served on the staff of both hospitals, and was an eminent citizen. His death occurred March 27, 1886.

The first lectures of the college were delivered at the Buffalo Hospital of the Sisters of Charity and later the Young Men's Christian Association building was utilized for that purpose. In 1884, the present college building located on Ellicott street, between Broadway and Clinton, was constructed and made ready for occupation about January 1, 1885. In 1891, this building was enlarged to its present proportions to meet the increasing demand for enlarged laboratories and ampler lecture rooms. An alumni association was organized in 1886, consisting of the faculty and lecturers of the college together with the graduates of that year. The officers were as follows: President, Dr. William H. Heath; first vice-president, Dr. R. B. Parks, Jamestown; second vice-president, Dr. E. J. Murphy, Buffalo; secretary, Dr. Geo. W. T. Lewis, Buffalo; treasurer, Dr. Simeon T. Clark, Lockport; executive committee, Drs. F. S. Crego, S. T. Clark and Anthony Hill, Buffalo.

The first commencement exercises of the college were held at Association Hall on the evening of April 12, 1886. The degree of doctor of medicine was conferred upon the following named candidates: E. J. Murphy, Buffalo; R. B. Parks, Jamestown; Thomas

Hill, Buffalo; George Wetherell, Toronto, and George W. T. Lewis, Buffalo; Anthony Hill, Buffalo.

The manner of conferring degrees at this institution is by "hooding," in pursuance of an ancient rite observed in many of

MEDICAL DEPARTMENT—NIAGARA UNIVERSITY.

the English universities. It is conducted as follows: The candidates, wearing their long black gowns, are introduced by a graduate to the chancellor of the university, with the following words:

*Insignissime Cancellarie: Presento tibi huncce scholarum in facultate medicinæ ut admittatur in gradum doctoris medicina testorque eum quoad omnia quae statuta requirunt aptum et idoneum esse.*

Each candidate then kneels before the chancellor, who holds the candidate's hands in his and, while the candidate is "hooded" by another graduate, who acts as a beadle, pronounces the following formula:

*Ad profectum reipublicae ego, auctoritate mea et totius universitatis admitto te ad gradum doctoris in medicina licentiamque tibi do omnia ea facienda quae ad illum gradum pertinent.*

The first address to the graduates was delivered by Dr. Simeon T. Clark, of Lockport, now deceased, professor of medical jurisprudence, who had been appointed to that chair, vice Hon. Joseph M. Congdon resigned. Dr. Clark, a gifted and versatile man, was seized of apoplexy while in the performance of his busy professional duties and died in the midst of a useful life, December 24, 1891.

The first public meeting of the Alumni Association was held April 12, 1887, at which Dr. William H. Heath presided. Papers were read at this meeting by Drs. Stephen Smith, of New York, and B. H. Daggett, Henry D. Ingraham and Frank Hamilton Potter, of Buffalo. The first public banquet which followed was served at the Genesee, at which seventy guests participated, including the faculty, alumni and invited guests.

The officers for 1895–'96 are: President, Joseph J. Kane, A. M., M. D.; first vice-president, Bentley S. Bourne, M. D.; second vice-president, Daniel F. White, M. D.; secretary, Henry Osthues, M. D.; permanent secretary, John J. Twohey, M. D.; treasurer, Frederick M. Boyle, M. D.; executive committee, James J. Mooney, M. D., Frederick A. Hayes, M. D., Joseph J. Finerty, M. D., all of Buffalo, N. Y. The association holds its annual meetings at the college hall on the commencement day of the medical school.

When this college was organised, two years' study in medicine was the legal requirement, but Niagara University, from the outset, insisted upon three years, while it recommended a four years' course. From the start it took a high stand and has been prosperously conducted by its able projectors since it was founded.

A law took effect September 1, 1891, establishing three years as the minimum course of medical study in the State of New York and establishing a separate state examining and licensing board, so that now all the medical colleges of the state are compelled by law to have the same minimum requirements for graduation.

The present faculty of Niagara University is constituted as follows: John Cronyn, M. D., Ph. D. LL. D., professor of principles and practice of medicine and clinical medicine, president of

BUFFALO HOSPITAL OF THE SISTERS OF CHARITY—1848–1876.

the faculty; Thomas Lothrop, M. D., Ph. D., professor of obstetrics, vice-president and treasurer of the faculty; Alvin Allace Hubbell, M. D., Ph. D., professor of ophthalmology and otology, secretary of the faculty; Henry D. Ingraham, M. D., professor of gynecology and pediatrics; William H. Pitt, M. D., Ph. D., professor of general chemistry and physics; Herman Mynter, M. D., professor of operative and clinical surgery; Herbert Mickle, M. D., professor of principles and practice of surgery; Carlton C. Frederick, M. D., adjunct professor of obstetrics; John A. Miller, M. Sc., A. M., Ph. D., professor of medical chemistry and toxicology; John D. Flagg, M. D., professor of physiology and microscopy; Eugene A. Smith, M. D., professor of general, descriptive and surgical anatomy; Henry C. Buswell, M. D., adjunct professor of principles and practice of medicine; William C. Krauss, M. D., professor of nervous diseases; L. Bradley Dorr, M. D., professor of bacteriology; W. Scott Renner, M. D., professor of laryngology; Walter D. Greene, M. D., professor of hygiene; Rollin L. Banta, M. D., professor of materia medica and therapeutics; Harry A. Wood, M. D., professor of insanity and adjunct professor of materia medica; Harlow C. Curtiss, A. M., professor of medical jurisprudence; Sidney A. Dunham, M. D., lecturer on physiology; Edward M. Dooley, M. D., adjunct professor of anatomy; Frederick A. Hayes, M. D., demonstrator of anatomy; Frederick Preiss, M. D., assistant in the principles and practice of surgery; David L. Redmond, M. D., lecturer on dermatology; Earl P. Lothrop, M. D., lecturer on pathology and clinical assistant in obstetrics; Alfred E. Diehl, M. D., lecturer on histology; George Roberts, M. D., lecturer on chemistry and demonstrator in pathology and bacteriology; Robert A. Poynton, M. D., lecturer on anatomy; Henry Osthues, M. D., Max Keiser, M. D., assistant demonstrators of anatomy.

Clinical assistants: Surgery—Frederick A. Hayes, M. D., Frederick Preiss, M. D., Henry Osthues, M. D. Medicine—R. L. Lounsberry, M. D., Alois Jokl, M. D., Max Keiser, M. D. Diseases of women, eye, ear and throat—John H. Daniels, M. D.

In concluding this sketch of the medical colleges, the suggestion is offered that, in view of the fact that the American Association of Medical Colleges has adopted a four years' course of study as a minimum requirement, and further, in view of the strong probability that soon a state law will be enacted on the subject, now is an appropriate time for Buffalo medical institutions to raise their curricula to four years' graded courses of collegiate study.

BUFFALO HOSPITAL OF THE SISTERS OF CHARITY—1895.

### III.—Hospitals.

### I.—BUFFALO HOSPITAL OF THE SISTERS OF CHARITY.

Although the subject had been many times agitated, it was not until 1848 that a hospital was really opened for the reception of patients in the city of Buffalo. A building located on what is now known as Pearl Place, made up of a group of several contiguous dwelling houses that had been previously occupied as an orphan asylum, was converted into a hospital and placed under the management of the Sisters of Charity. This building is now occupied as a tenement. It was incorporated under the laws of the State of New York and accommodations were provided for 100 patients. Later an appropriation of $9,000 was made by the state. It was in readiness by August, 1848, and during the first six months 121 patients were received. It was provided that no questions should be asked of the patients when admitted touching matters of religion, and that applications for admission should be made to the medical board, to the president of the Good Samaritan Society, or of the Society of St. Vincent of Paul and, further, that a line from the pastor of any church of any denomination should also secure admission. The following was the schedule of weekly prices :

1. For the sick-poor in the general ward to include board, washing, medical attendance, nursing and medicine, $1.50.
2. For those in the marine ward the rate fixed by government.
3. For the sick who can pay for board, medicine, attendance, washing, and the like in a general ward, $2.50.
4. For the sick who are able to pay and who desire private rooms, $4.

The first medical board was constituted as follows : Frank H. Hamilton, M. D., attending surgeon ; Austin Flint, M. D., attending physician ; James P. White, M. D., consulting surgeon ; Josiah Trowbridge, M. D., consulting physician.

Appreciating the importance of clinical instruction, the late Bishop Timon, a learned prelate of the Roman Catholic Church, threw open the doors of the hospital for that purpose, where, for a small fee, the students of the medical college, then lately established, received bedside training under the supervision of an attending physician or surgeon.

During the cholera epidemic of 1849 there were admitted into this institution previous to September 1st, 136 patients suffering with this disease, fifty-two of whom died. This evidently was a busy year for the hospital, as the report for that year, made November 27th, shows that 1,513 patients in all were admitted, of whom more than one-half were charity cases. The records of the first fourteen years were destroyed by fire, so it is impossible to trace its interesting history during that period.

From time to time the capacity of the hospital was increased, so that finally it could accommodate nearly 200 patients. It, however, soon outgrew its first location and in 1872 a site was purchased on North Main street, corner of Delavan avenue, for a new and larger hospital building. In June, 1875, ground was broken, in August the corner-stone was laid and on November 5, 1876, the hospital was dedicated. The cost of the buildings and ground was $168,368.

This is a large, substantial, four-story brick structure with basement situated upon high ground surrounded by beautiful broad lawns. When the new wing now building is completed the hospital will be as represented in the illustration given on page 93. It is a modern building with all the conveniences necessary for its numerous patients, and when finished it will have its own electric plant for lighting, and it will be heated and ventilated according to the latest and best methods. One of the best arranged and most complete surgical operating rooms in the state is in this hospital. The new gynecological operating room will be equally as complete as the surgical. The cost of the building when completed will be about $250,000 and its capacity will be 344 beds. In addition a contagious pavilion, containing from twenty-five to thirty beds, will be erected during the present summer.

This was one of the first hospitals in the United States under the management of the Sisters of Charity to establish the custom of resident physicians, and it was the first one under the Sisters' management to establish a training school for nurses.

The present medical staff is composed of the several professors of Niagara University who have charge in their respective branches.

### II.—BUFFALO GENERAL HOSPITAL.

Meetings of several citizens were held at the office of Henry W. Rogers, collector of the port of Buffalo, on the 23d and 26th of October, 1846, at which an association was formed for the establishment of a public hospital in this city. Thirty-five directors were appointed and officers were elected as follows: President, Josiah Trowbridge, M. D.; first vice-president, Gen. H. B. Potter; second vice-president, Geo. W. Clinton; secretary, E. S. Baldwin; treasurer, S. N. Callender; executive committee, R. H. Heywood, Bryant Burwell, M. D., and George Jones. Dr. F. H. Hamilton was appointed attending surgeon, and Dr. Austin Flint, attending physician, with Drs. Trowbridge and Burwell respectively as consulting physician and surgeon.

It was subsequently announced that a building known as the Seamen's Home had been obtained temporarily, to be used as a city hospital. This organization seems to have disappeared.

BUFFALO GENERAL HOSPITAL—1858.

Opposition was met with and an appropriation which was nearly obtained from the state was lost. The necessity for a hospital was great, but the next year the Buffalo Hospital of the Sisters of Charity went into operation, which met the existing emergency. The rapid growth of the city, however, soon made another hospital necessary; hence, in 1854, a second attempt was made with Millard Fillmore at the head of the board of trustees, which consisted of fifty members. It was thought unadvisable to commence operations without a capital of $100,000, but as the money could not be raised, this project, too, was abandoned. Finally, a board of nine trustees was appointed, consisting of Charles E. Clark, president; Andrew J. Rich, vice-president; William T. Wardwell, secretary and treasurer, and George S. Hazard, Bronson C. Rumsey, Roswell

L. Burrows, Stephen C. Howell and Henry Martin. On the 21st of November, 1855, the association was formed and the certificate thereof was filed in the county clerk's office, December 13, 1855. The sum of $20,000 was subscribed by citizens and in 1857 the hospital received an appropriation from the state of $10,000. A building was erected on High street on a site which was considered

BUFFALO GENERAL HOSPITAL AS ENLARGED, 1880.

one of the finest in the city, having 361 feet front on High, 450 feet on Goodrich street and a depth of 282 feet. The west wing of the building was rapidly pushed to completion and was dedicated June 26, 1858, with appropriate ceremonies amidst an enthusiastic gathering of citizens. An address was delivered by the Hon. James O. Putnam, which closed with the following outburst of lofty eloquence:

Citizens of Buffalo—The offering we this day dedicate is yours, to cherish and to place upon an enduring basis. It is one of the noblest that can be brought into the temple of humanity. That temple is wide as the heavens and receives within its portals every child of affliction and sorrow. That charity which came to earth an angel, attendant upon the Babe of Bethlehem, knows no distinction of caste, complexion or nationality. She asks not at what altar the sufferer worships, and before she relieves, does not stop to inquire whether he even be a worshiper at all. And if she chance to find him without a faith and without a God, poor in soul as he is wretched in body, she delights, so far as comports with delicacy and propriety, in the double office of ministering to his temporal necessities, while with gentle guidance she points the wanderer 'to brighter worlds and leads the way.' I seem to hear a voice coming up through the vale of the centuries, clear and resonant, 'go and do thou likewise.'

Thus the third attempt to establish a general hospital proved successful.

The following-named physicians were appointed medical officers for one year dating from July 1, 1858: Attending physicians, Drs. James M. Newman, Thomas F. Rochester and C. C. Wyckoff; consulting physicians, Drs. James P. White, George N. Burwell and P. H. Strong; attending surgeons, Drs. Charles H. Wilcox, Sanford Eastman and Austin Flint, Jr.; consulting surgeons, Drs. Frank H. Hamilton, C. C. F. Gay and John Root. Dr. Walter B. Coventry was the first resident physician.

A new wing was afterward erected that was dedicated October 1, 1880, bringing the present capacity of the hospital up to 150 beds. A training school for nurses was instituted about this time that has been in successful operation ever since. A nurses' home has been built on the hospital grounds.

The demands of a largely increased growth of the city are such as to overflow the accommodations of all our charities. A further enlargement of the hospital is, therefore, contemplated on lines that are already drawn by the architect, an allusion to which was made in the April, 1895, issue of the JOURNAL, page 554. The munificent gift of $55,000 made by Mrs. George B. Gates and her three daughters, Mrs. William Hamlin, Mrs. Charles W. Pardee and Miss Elizabeth Gates, renders it possible to begin this work at once. When completed it will be one of the most substantial and beautiful hospital structures in the country.

The hospital staff is at present constituted as follows: Consulting physicians, Cornelius C. Wyckoff, M. D., Conrad Diehl, M. D., M. B. Folwell, M. D.; consulting surgeons,

PROPOSED BUFFALO GENERAL HOSPITAL.

From *The Illustrated Buffalo Express* Copyrighted, 1895, by Geo. E. Matthews & Co.

John Hauenstein, M. D., Devillo W. Harrington, M. D.; consulting obstetrician, Matthew D. Mann, M. D.; consulting neurologist, James W. Putnam, M. D.; consulting aural surgeon, William C. Barrett, M. D.; consulting dermatologist, Ernest Wende, M. D.; attending physicians, Charles Cary, M. D., Henry R. Hopkins, M. D., Charles G. Stockton, M. D.

Attending surgeons, Roswell Park, M. D., and William C. Phelps, M. D.; gynecologist, Matthew D. Mann, M. D.; obstetrician, P. W. Van Peyma, M. D.; pathologist, Herbert U. Williams, M. D.; ophthalmic and aural surgeons, Frank W. Abbott, M. D., and Lucien Howe, M. D.

Assistants to medical staff: DeLancey Rochester, M. D., assistant physician; M. A. Crockett, M. D., assistant gynecologist; John Parmenter, M. D., assistant surgeon.

Resident staff: Edward F. Horr, M. D., Sidney D. Wilgus, M. D., P. R. Outlaw, M. D., Grant Cooper, M. D., Kate Isabel Kennedy, superintendent of nurses.

### III.—BUFFALO STATE HOSPITAL.

The corner-stone of this institution was laid September 18, 1872, with masonic ceremonies in the presence of a large number of citizens. Governor John T. Hoffman pronounced an address, Dr. James P. White, president of the board of managers, made some introductory remarks and the Hon. James O. Putnam delivered a formal address. The commissioners designated to locate the hospital in Western New York were appointed in 1869 by Gov. Hoffman, namely—Dr. John P. Gray, Utica; Dr. James P. White, Buffalo; Dr. Thomas D. Strong, Westfield; Dr. William B. Gould, Lockport, and Dr. Milan Baker, Warsaw. The first board of managers was as follows: Dr. John P. Gray, Utica; Asher P. Nichols, Buffalo; Dr. William B. Gould, Lockport; Lorenzo Morris, Fredonia; Augustus Frank, Warsaw; Albert P. Laning, William G. Fargo, George R. Yaw, Dr. James P. White and Joseph Warren, Buffalo. It is appropriate to state that it was chiefly due to the efforts of Dr. White that the hospital was located in Buffalo. The erection of the administration building and the east wing was proceeded with at once. It was not, however, until 1880 that it was made ready for the reception of patients. Dr. Judson B. Andrews, of Utica, was appointed superintendent, and under his able management the hospital soon assumed a leading position among institutions for the care of the insane in this country.

The work on the west wing was begun in 1889 and the first building was completed in 1891. The second building was completed in 1895 and the three remaining buildings are now in pro-

JUDSON BOARDMAN ANDREWS, M. D.

cess of erection, which will make the westerly wing symmetrical with that of the easterly side of the center building.

A training school for nurses was established about ten years ago, this being the first public institution for the treatment of the insane to establish such a school in this country. The school has over 100 graduates, many of whom are doing private nursing successfully in this city and sections of the state. The hospital also has a nurses' home situated upon the grounds.

There is in contemplation the erection of an infirmary building for acute cases, which will be complete in every respect with laboratories, operating rooms and isolated wards for the acutely sick. The institution now has a population of 841, and when the new buildings are completed the capacity of the hospital will be about 1,200. The cost of the building, when the present wing is completed, will be approximately $2,000,000.

The following-named gentlemen compose the present board of managers: Dr. John Cronyn, president, Hon. Daniel H. McMillan, Charlotte S. Williams, Alphonse J. Roehner, Dr. Thomas Lothrop, Joseph P. Dudley, Charlotte T. M. Glenny, Dr. Roswell Park, Buffalo; Hon. John E. Pound, Lockport; Frederick P. Hall, Jamestown.

The following-named are the present medical officers: Dr. Arthur W. Hurd, superintendent, Drs. Percy Bryant, Herman G. Matzinger, George G. Armstrong, Walter H. Conley, Helene Kuhlmann and Walter H. Kidder, assistant physicians.

### IV.—PROVIDENCE RETREAT.

The Providence Retreat is a private institution for the care and treatment of the insane, conducted by the Sisters of Charity. It was opened July 15, 1861, and now has a capacity for 175 patients. Dr. William Ring was the first attending physician, and the physicians now in charge are Drs. Floyd S. Crego and Harry A. Wood. The consulting physicians are Drs. John Cronyn, Conrad Diehl, Thomas Lothrop, E. C. W. O'Brien, J. W. Putnam and Ernest Wende; consulting surgeon, Dr. Herman Mynter; consulting gynecologist, Dr. Henry D. Ingraham; consulting oculist, Dr. A. A. Hubbell.

### V.—ERIE COUNTY HOSPITAL.

The law regarding the state care of the insane that took effect in 1893 left vacant the commodious and substantial structure that had been used by the county as an insane hospital. Recognising the desirability as well as economy of using this building as a hospital for the county sick, a number of physicians, under the leadership of Dr. John H. Pryor, brought this subject to the notice of

BUFFALO STATE HOSPITAL.

the board of supervisors, when, finally, the Erie county hospital was created and a visiting and consulting staff appointed. It was organised about January 1, 1894, and is situated on North Main street near the city line. The capacity of the hospital at present is 360 beds, the average population being 325 patients, but as many as 388 have been accommodated at one time.

A new consumption hospital annex with a capacity for eighty patients is in course of construction. This building is separated from the main structure, and the theory that consumption is an infectious disease pervades the entire principles of its erection.

The medical staff itself proposes to supply the consumption annex with all comforts, including billiard tables and other means of entertainment, and it is believed that this will be the first consumption hospital built in the state.

The Erie County Hospital has a training school of thirty-two nurses under the management of Miss Sarah Bond Lowe, who is assisted by five graduated nurses, each ward being placed under the supervision of a trained nurse.

The hospital staff is made up as follows: Consulting physicians, Drs. Thos. Lothrop, Chas. G. Stockton, N. Osborne; consulting surgeons, Drs. Roswell Park, Herman Mynter, Claudius Niemand; attending physicians, Drs. Jno. H. Pryor, DeLancey Rochester; attending surgeons, Drs. W. S. Tremaine, John Parmenter, Eugene Smith, Dewitt G. Wilcox; gynecologists, Drs. Herman E. Hayd, Stephen Y. Howell, Carlton C. Frederick, George T. Mosely; obstetricians, Drs. P. W. Van Peyma, Rollin L. Banta; diseases of eye and ear, Drs. A. A. Hubbell, Elmer E. Starr, F. Park Lewis; diseases of nose and throat, Dr. W. Scott Renner; genito-urinary surgery, Dr. William H. Heath; diseases of skin, Dr. Grover W. Wende; orthopedic surgery, Dr. Bernard Bartow; diseases of nervous system, Drs. Wm. C. Krauss, James W. Putnam; diseases of rectum, Dr. Edward Clark; pathology, Drs. Herbert U. Williams, Francis T. Metcalfe. Officers of the medical staff, Dr. John H. Pryor, president; Dr. Francis T. Metcalfe, secretary; executive committee, Drs. John H. Pryor, F. Park Lewis, W. S. Tremaine, P. W. Van Peyma, Edward Clark, H. E. Hayd, F. T. Metcalfe. House staff, Dr. E. J. Gilray, medical superintendent; resident physicians, Drs. Chas. Helvie, H. L. Bender, L. B. Lockard, Geo. Mord, Wm. House, Marshall Clinton, J. H. Robinson; superintendent of nurses, Miss Sarah Bond Lowe.

### VI.—BUFFALO WOMAN'S HOSPITAL.

This hospital was established by Dr. Thomas Lothrop in May, 1886, to receive and care for women, married or single, during childbirth or while suffering from diseases peculiar to their sex. At first it was located on the corner of Seventh and Maryland streets, but in May, 1891, it was removed to its present situation, 191 Georgia street, corner Seventh, where it occupies a large and well-appointed

BUFFALO WOMAN'S HOSPITAL.

building. It receives a limited number of worthy indigent women suffering from curable disease, free of expense, provided that they are unable to pay for their board and treatment. There is also a free dispensary maintained in connection with the hospital. The private rooms are suitably furnished and supplied with all comforts consistent with the necessities of modern surgical cleanliness. It is in this hospital that the pupils of Niagara University receive such admirable obstetric training. Under the supervision of Professor Lothrop each member of the senior class is enabled to attend from three to six cases of labor before graduation. Dr. Thomas Lothrop is the physician-in-chief and Dr. C. C. Frederick is the surgeon-in-chief. Drs. Jacob H. Meyer, William G. Taylor and Earl P. Lothrop are clinical assistants.

The following-named gentlemen compose the consulting staff: Drs. W. S. Tremaine, Herman Mynter, Rollin L. Banta, Henry C. Buswell, Wm. Warren Potter, Herbert Mickle, Eugene A. Smith, Walter D. Green.

### VII.—ST. MARY'S ASYLUM FOR WIDOWS, FOUNDLINGS AND INFANTS.

This institution is located at 126 Edward street, and is under the charge of ten Sisters of Charity. It was opened June 1, 1854, with accommodations in two cottages for fifteen inmates; Dr. James P. White was the attending physician, and Sister Rosalie, with two other sisters, was in charge. It has at present accommodations for about 185 persons. After Dr. White's term of service expired Dr. James S. Smith took charge, and he was followed by Dr. Eugene A. Smith. Dr. Thomas Lothrop and Dr. C. C. Frederick are now in charge of the lying-in wards, and Dr. Earl P. Lothrop is clinical assistant. The present attending physician is Dr. Eugene A. Smith, who is assisted by Drs. Henry Osthues and B. H. Brady.

### VIII.—ST. FRANCIS ASYLUM.

This institution, located at 337 Pine street, was established December 18, 1861. The founder, Mother M. Francis Bachman, with three sisters of the Franciscan order, came from Philadelphia, where they had established a similar asylum. It has for its object the care of aged poor of both sexes, regardless of nationality or religious denomination. The average number of inmates, from 1863 to 1867, was nineteen; during the past ten years the average was 245. At present there are 300 inmates in the institution. The number of sisters in attendance is thirty-two. The total number of Franciscan sisters is 170, who are engaged in the various institutions of the order located in different cities.

Formerly Drs. Edward Storck and Conrad Diehl were attending physicians. Now Drs. Thomas Lothrop, John D. Flagg, William C. Krauss and A. E. Persons compose the attending staff.

ERIE COUNTY HOSPITAL.

### IX.—BUFFALO CHILDREN'S HOSPITAL.

This institution was established in September, 1892, through the generosity of Mrs. Gibson T. Williams and Miss Martha Williams, who purchased the property, 219 Bryant street, and after refitting it offered it rent free to the board of managers, which is composed of a group of philanthropic women. The hospital now has accommodations for thirty-six patients.

The following is the present list of officers: Mrs. Lester Wheeler, president; Mrs. George H. Lewis, vice-president; Mrs. Henry Watson, Mrs. Bainbridge Folwell, purveyors; Miss Martha T. Williams, treasurer; Mrs. Bernard Bartow, secretary.

Executive committee: Mistresses E. B. Alward, George Truscott, S. S. Spaulding, Wm. Hamlin, Henry Bull, T. T. Ramsdell, Nathaniel Rochester, Dexter P. Rumsey, George Parkhurst, Edwin Bell, E. P. Fish, Chas. Pardee, John L. Williams, Joseph Hunsicker.

Advisory committee: Messrs. George H. Lewis, Sherman S. Rogers, G. L. Williams, C. Sidney Shepard, Henry W. Sprague.

The medical staff is as follows: Attending physician, Dr. Bainbridge Folwell; attending surgeon, Dr. John Parmenter; orthopedic surgeon, Dr. Bernard Bartow; assistant physician, Dr. Dewitt H. Sherman; assistant surgeon, Dr. Loren H. Staples; neurologist, Dr. Chas. S. Jones; ophthalmic and aural surgeon, Dr. H. Y. Grant, and nose and throat surgeon, Dr. W. Scott Renner.

### X.—BUFFALO HOMEOPATHIC HOSPITAL.

The Buffalo Homeopathic Hospital was organised June 14, 1872, and is located at 74 Cottage street, corner Maryland. We are unable to give the names of the first medical staff, but the board of trustees for the first year was made up as follows: * Jerome Pierce, * Chas. C. McDonald, * Benj. H. Austin, Sr., Loran L. Lewis, * Jas. Brayley, * Francis H. Root, * Jerome F. Fargo, * John B. Griffin, Samuel V. Parsons, * Mrs. C. C. Warner, * Mrs. M. A. Kenyon, * Mrs. Hannah Fargo, Mrs. Anna Poole Hoxsie, Mrs. Hattie E. Gregg, Mrs. Charlotte E. Lewis. The capacity of the hospital is about sixty patients.

The medical and surgical staff now in service is as follows: President, Dr. A. M. Curtiss; first vice-president, Dr. Henry Baethig; second vice-president, Dr. H. A. Foster; secretary, Dr. Geo. T. Moseley.

---

* Deceased.

CONSUMPTION ANNEX—ERIE COUNTY HOSPITAL.

Consulting physicians: Drs. A. R. Wright, A. T. Bull, H. A. Foster, D. B. Stumpf, H. Baethig, N. Osborne, A. M. Curtiss, John Miller.

Attending physicians: Drs. E. P. Hussey, B. J. Maycock, E. A. Fisher, J. T. Cook, T. J. Martin, C. S. Albertson.

Attending surgeons: Drs. H. C. Frost, D. G. Wilcox, G. T. Moseley; obstetricians, Drs. J. S. Halbert, G. R. Stearns; ophthalmic surgeon, Dr. F. Park Lewis; pathologist, Dr. A. W. Dods; pharmacist, Dr. P. A. McCrea.

Junior staff physicians: Drs. A. B. Eadie, E. Bodenbender, N. Bodenbender, D. Schladermundt, P. L. Carter.

Junior staff surgeons: Drs. M. F. Linquist, M. Manges, W. H. Marcy, H. L. Towner; ophthalmologists, Drs. W. A. M. Hadley, F. D. Lewis; obstetricians, Drs. Jessie Shepard, Rose Wilder; laryngologist, Dr. F. L. Barnum.

### XI.—LEXINGTON HEIGHTS HOSPITAL.

This is a private hospital under homeopathic management. It was established May, 1890, as the Wilcox Private Hospital, under which name it was conducted for two years. A stock company was then formed, called the Wilcox Hospital Company, which continued in control for one year. It was converted into the Buffalo Hospital Company in 1893, since which time it has been conducted under the name of the Lexington Heights Hospital. It receives obstetrical, surgical and general patients. Dr. Dewitt G. Wilcox is the president of the company as well as the surgeon in charge. Associated with him as a medical staff are the following-named gentlemen, who attend patients in their respective branches: George T. Moseley, M. D., gynecologist; Charles S. Albertson, M. D., obstetrician; Philip A. McCrea, M. D., skin diseases; Monroe Manges, M. D., assistant surgeon; Nehemiah Osborne, M. D., diseases of kidneys; Burt J. Maycock, M. D., heart and lungs; William C. Krauss, M. D., neurologist; H. L. Towner, M. D., orificial surgeon; S. A. Dunham, M. D., alcoholism and dipsomania; P. Livingston Carter, M. D., diseases of the stomach; A. B. Eadie; M. D., diseases of children; Wm. A. M. Hadley, M. D., oculist and aurist; Fred D. Lewis, M. D., laryngologist; Nelson Bodenbender, M. D., Edward Bodenbender, M. D., Chas. A. Schladermundt, M. D.; J. Jay Cook, dentist.

Besides these there is an emergency hospital, located on the corner of South Division and Michigan streets, which is an annex

VIEW OF BUFFALO IN 1895.

From the original engraving, 43 x 26 inches, first published in the *Illustrated Buffalo Express*, April 14, 1895.

of the Buffalo Hospital of the Sisters of Charity ; the Fitch accident hospital, corner of Swan and Michigan streets, a department of the Charity Organization Society ; the Riverside Hospital for Women, 2393 Niagara street, under the charge of Dr. Lillian C. Randall ; and the Fresh Air Mission hospital, at Athol Springs. There are a number of eye and ear infirmaries and dispensaries, an account of which we are obliged to omit for want of space. The Railroad Branch of the Young Men's Christian Association has a hospital located on Broadway near Bailey avenue which is intended for emergency cases.

In 1845, there were no public hospital accommodations ; in 1895, there is an aggregate of 3,000 beds.

### IV.—Medical Societies.

The Buffalo Medical Association was organized July 2, 1845, at the office of Dr. Josiah Trowbridge, who was chosen president; Dr. Alden S. Sprague, vice-president, and Dr. Austin Flint, recording secretary. Previous to this there had been two attempts to create a medical society in Buffalo, the first of which was made July 19, 1831, when the village contained about 9,000 inhabitants. Of this society Dr. Cyrenius Chapin was president, and its last meeting was held June 5, 1832.

The first meeting of the second organization was held January 22, 1836, but this proved even a more significant failure than the other.

The first regular meeting of the Buffalo medical association was held at the office of Dr. F. H. Hamilton, August 5, 1845, at 8 o'clock P. M. Dr. Flint presented for inspection a heart with valvular lesions; Dr. Hamilton moved the appointment of a committee to collect information concerning fractured limbs, shortening and the like, and at the September meeting Dr. White presented a placenta with ossific deposit and reported a case of ovarian dropsy, caused by a severe fall on the abdomen. We have mentioned the reports of these three men at this time because they, perhaps more than any others, gave direction to the efforts of the society; but especially would we call attention to the pathology of ovarian tumors as then taught by the distinguished obstetrician and gynecologist, who for thirty-five years gave force and impact to the profession of Buffalo not only in his own department of medicine, but in a larger and more general sense. This is in strange contrast with the pathology of today, and it is a pleasant thought that Dr. White was one of the first teachers to modify his earlier views on this as well as other subjects, always keeping pace with or leading progress, and lived to become one of the first abdominal surgeons of his time.

The only living foundation member of this society within our knowledge is Dr. James B. Samo, who is an honored member of the medical profession in Buffalo.

In 1856, the society was legally incorporated, its name changed to the Buffalo Medical and Surgical Association and a new code of by-laws was adopted which continued in force until June 3, 1879, when still another constitution and code of by-laws were put into operation. These governed until the

BUFFALO ACADEMY OF MEDICINE

was founded May 17, 1892. This latter organization was formed by grouping a number of associate societies under one administration, by which it was hoped to concentrate and make more cohesive the medical talent of the city. These societies were the Buffalo Medical and Surgical Association, which became the surgical section; the obstetrical society, that became the section on obstetrics and gynecology; the pathological society, that became the section on anatomy, physiology and pathology, and the clinical society, that became the section on medicine, materia medica and therapeutics.

The first officers of the academy, elected June 21, 1892, were: president, De Lancey Rochester, M. D.; secretary, William C. Krauss, M. D.; treasurer, Eugene A. Smith, M. D.; trustees, Drs. J. W. Putnam, A. Dagenais and Roswell Park.

The present officers are: President, Dr. Herman Mynter; first vice-president, Dr. W. S. Tremaine; second vice-president, Dr. Lucien Howe; third vice-president, Dr. H. U. Williams; fourth vice-president, Dr. C. C. Frederick; secretary, Dr. A. L. Benedict; treasurer, Dr. Eugene A. Smith; trustees, Drs. De Lancey Rochester, H. R. Hopkins and F. W. Bartlett.

Besides these there are several private medical societies, the oldest of which is the Medical Club that meets on alternate Wednesday evenings, and the Medical Union which meets the third Tuesday of every month. The Medical Society of the County of Erie meets in Buffalo, but as its constituency embraces the entire county it is out of place to enter into details of its history here. For a like reason we need only mention the Homeopathic Society of Erie County.

Finally, let it be remembered that Buffalo has now upwards of 360,000 inhabitants as contrasted with less than 30,000 when the first events occurred that are related in this narrative. We cannot accentuate the effect of this marvelous growth better than to call attention to the two views of the city that we publish. The first one—that of 1845—is from an old lithograph in possession of the historical society, reduced and engraved by Matthews, Northrup & Co., to which firm we are indebted for many courtesies in the preparation of this article. The view of Buffalo in 1895 is a triumph of art. It is a composite picture and the original, from which this is a reduction, is the largest of its kind ever made. Though Buffalo has grown rapidly, medicine has kept pace with the material increase of the city.

Before concluding this sketch it may not be out of place for the writer to remark that no one can be more conscious of its imperfections than himself. It does not, however, aim to be a complete history of medical affairs in Buffalo for the last fifty years; it would require a large volume to contain that. But it seeks—and with a truth, modestly—to give a résumé of the salient events of that period, and especially to group a mass of facts pertaining to the subject in the earlier years covered by the narrative. Again, it has been thought best to give by name those physicians who are now conducting the colleges, hospitals and societies in question, even at the risk of extending the article beyond the limits originally laid out. Finally, in the illustrations, we have again, so far as the buildings are concerned, sought to couple the past with the present, and, in some instances, to foreshadow the future, but in no instance have we thought it advisable to publish the portrait of a living individual. Had this rule not been adopted, it is doubtful if there had been an end to this article that is already too long.

A. R. DAVIDSON, M. D.

1879—1888.

FRANK H. POTTER, M. D.
Obit., July 16, '91.

F. R. CAMPBELL, M. D.

Obit., Sept.14,'88.

EDITOR AND ASSOCIATE EDITORS BUFFALO MEDICAL JOURNAL.

## Figures and Tables

**Figure f1:**
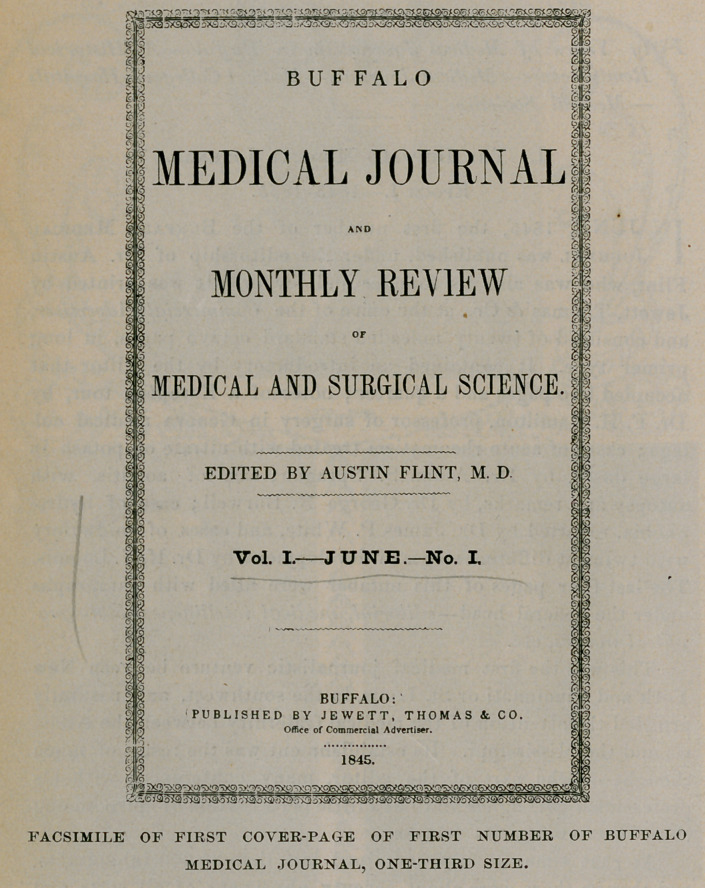


**Figure f2:**
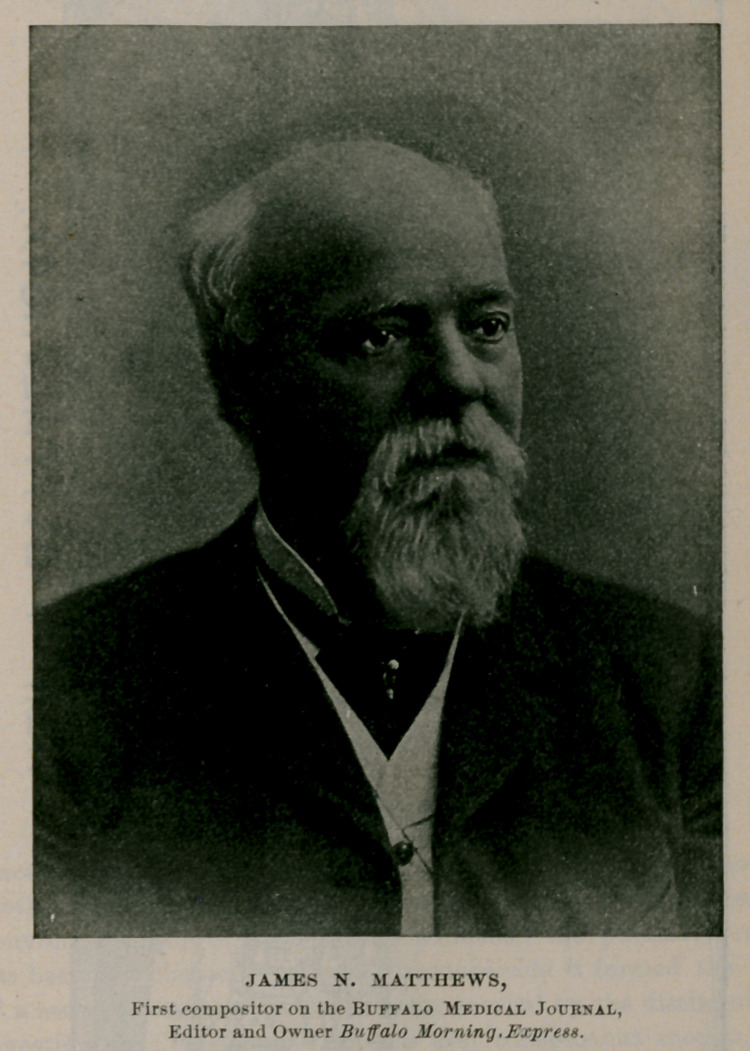


**Figure f3:**
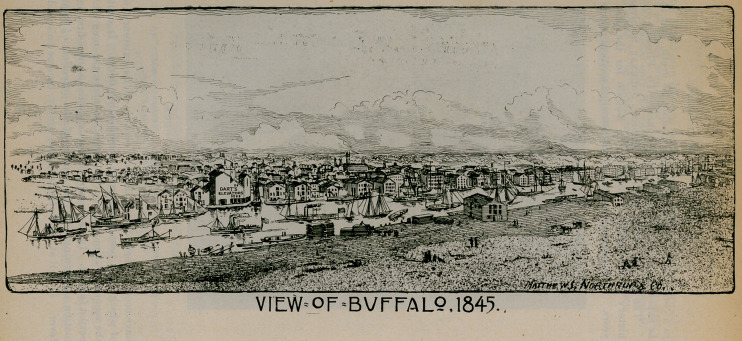


**Figure f4:**
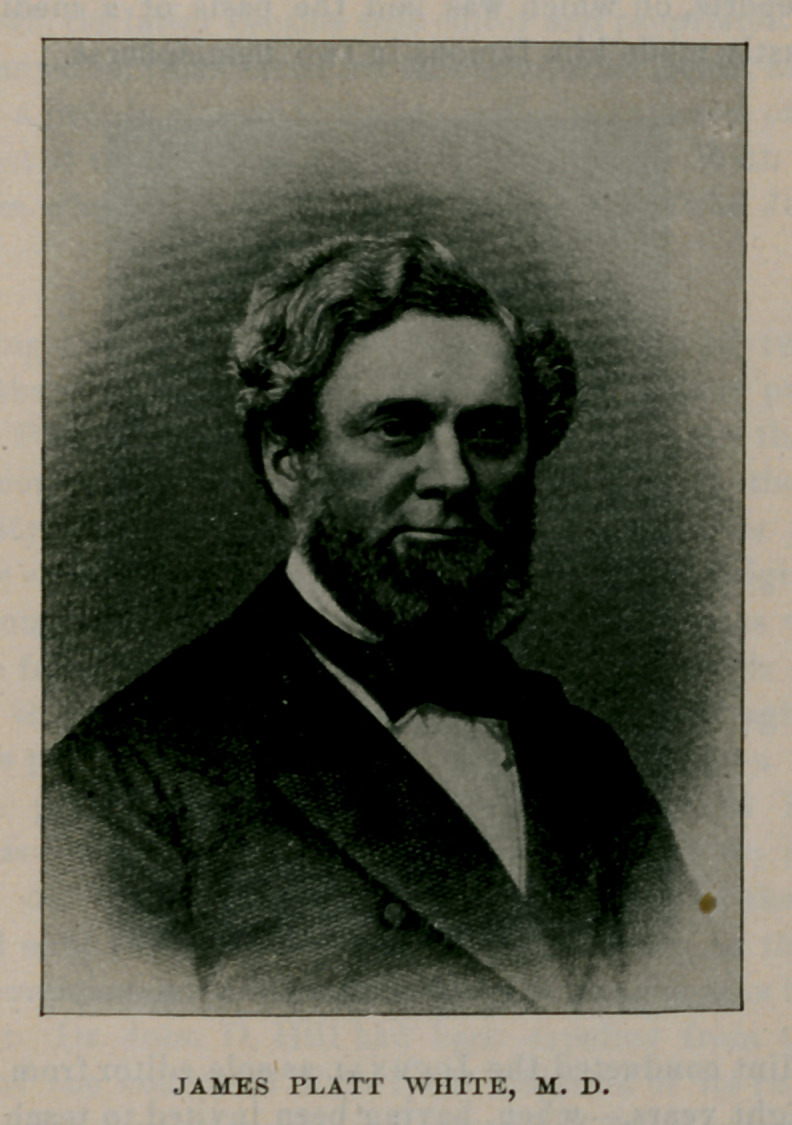


**Figure f5:**
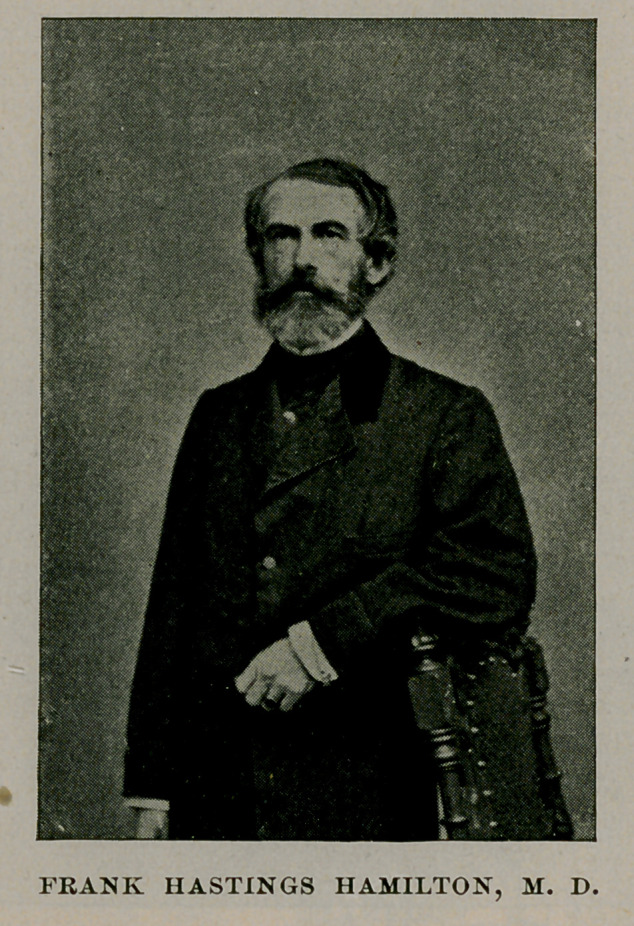


**Figure f6:**
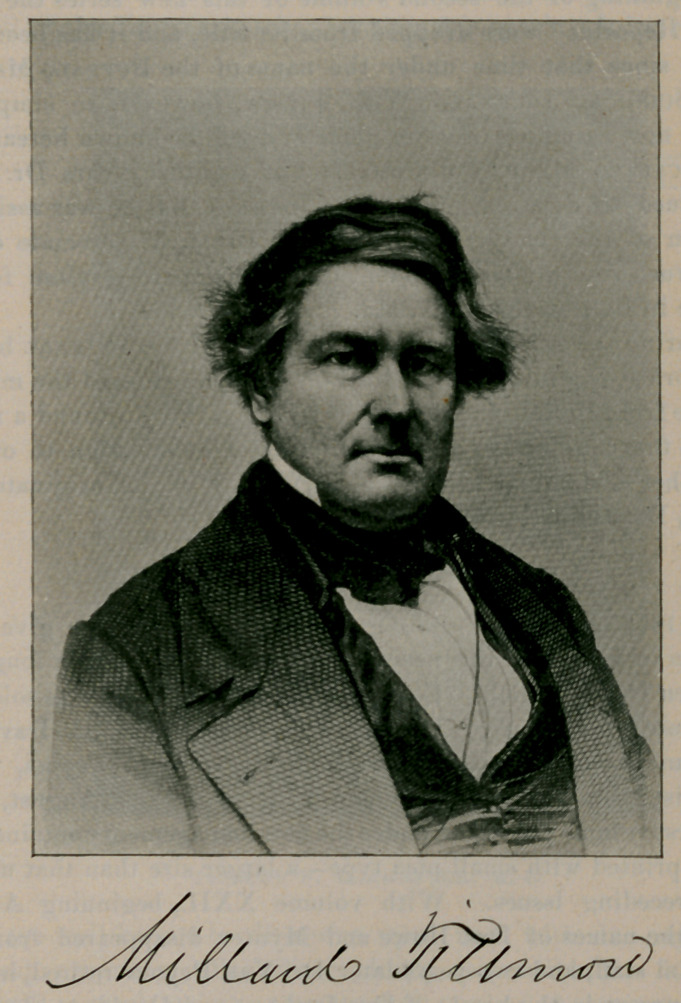


**Figure f7:**
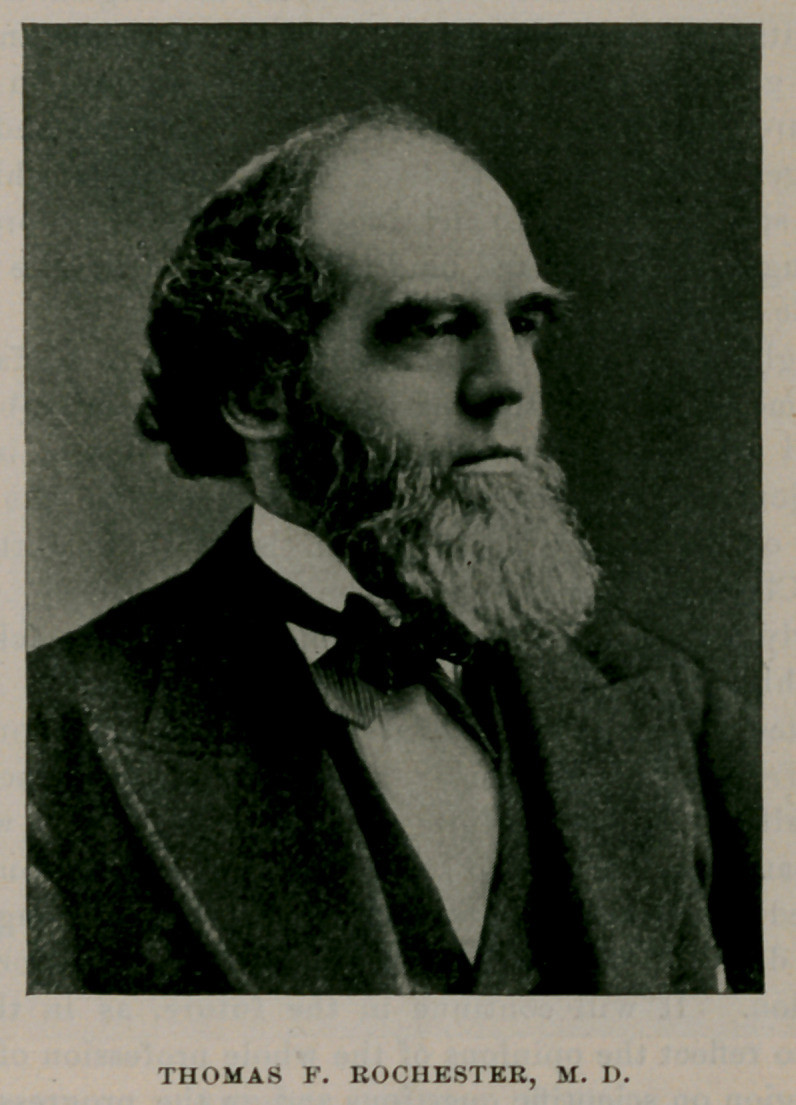


**Figure f8:**
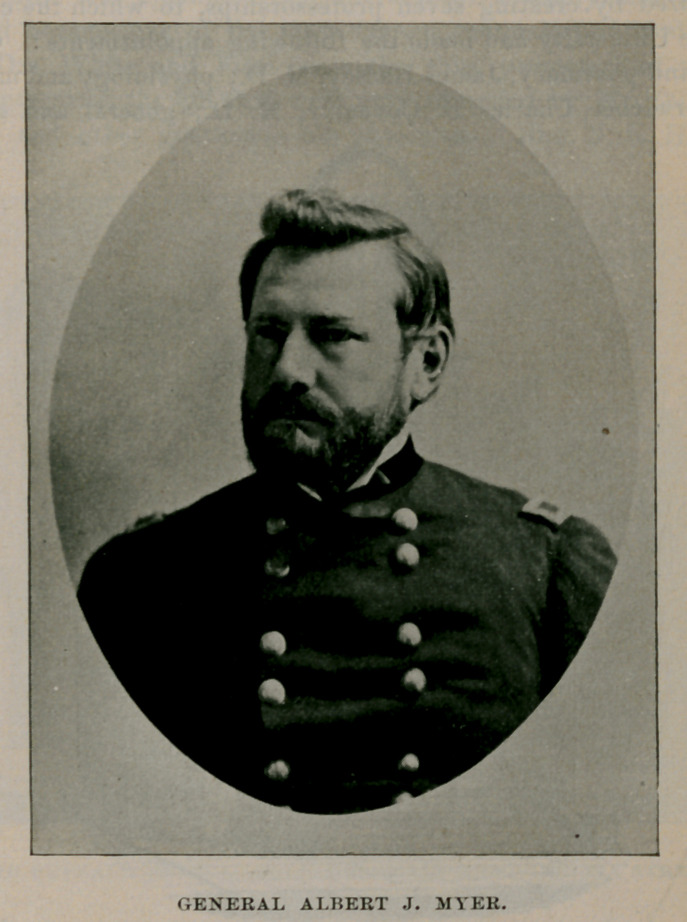


**Figure f9:**
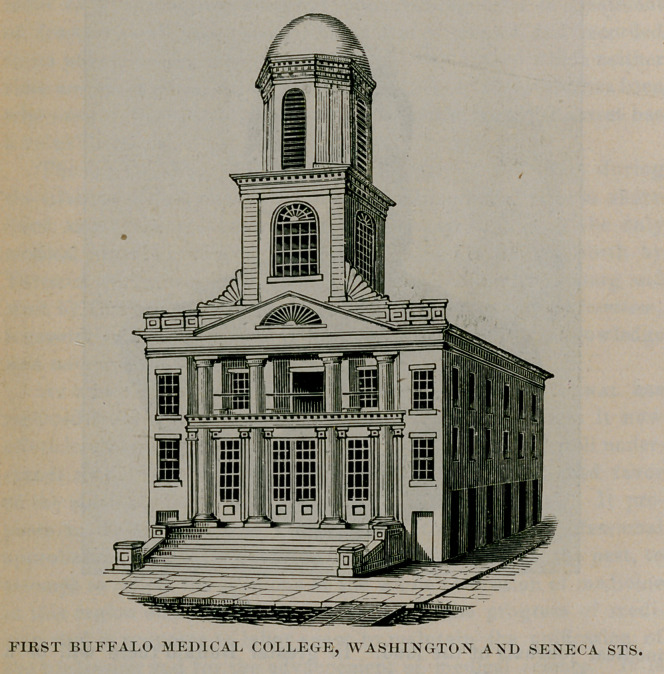


**Figure f10:**
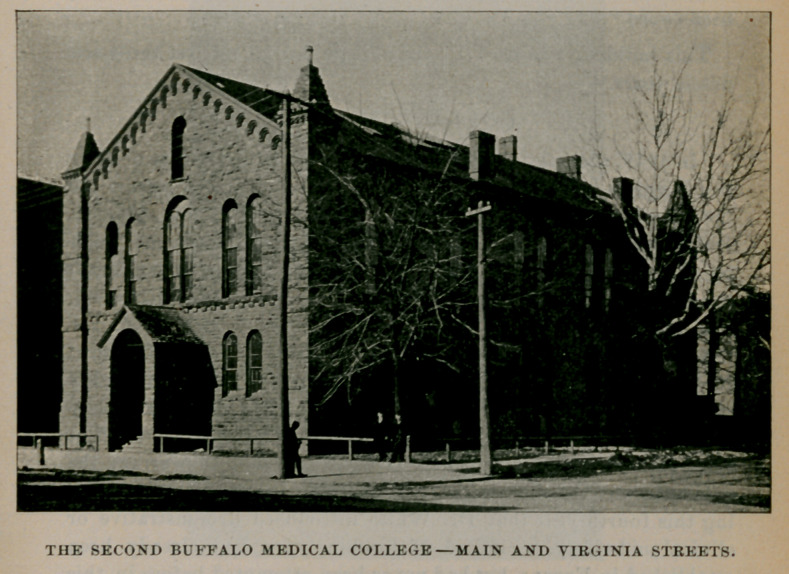


**Figure f11:**
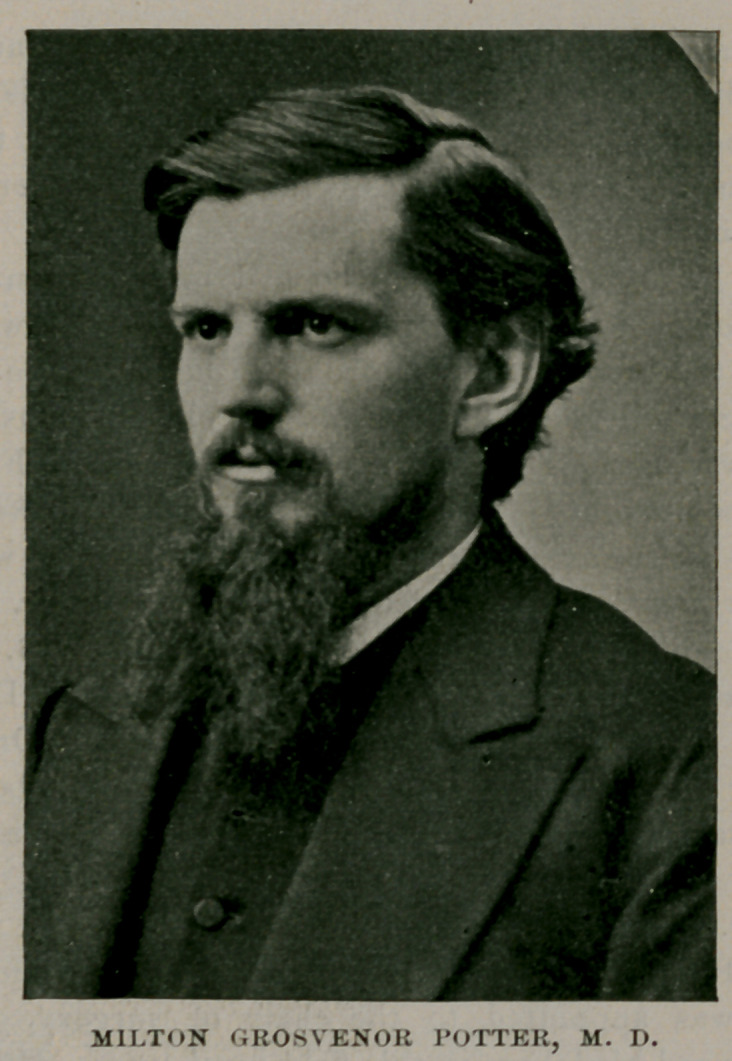


**Figure f12:**
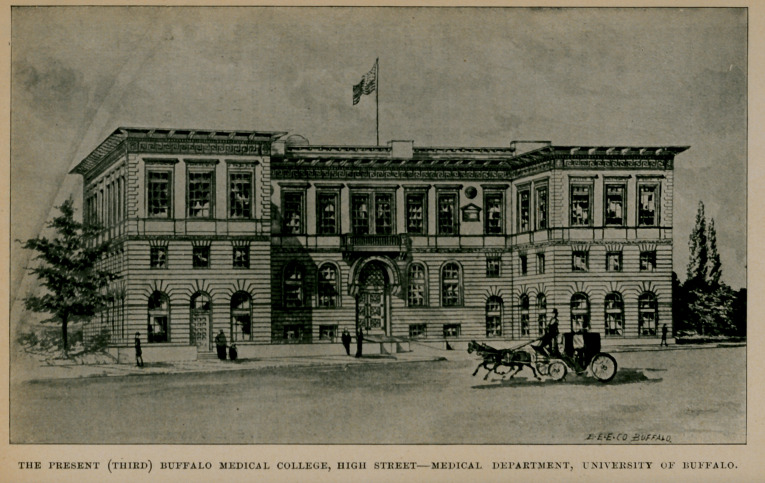


**Figure f13:**
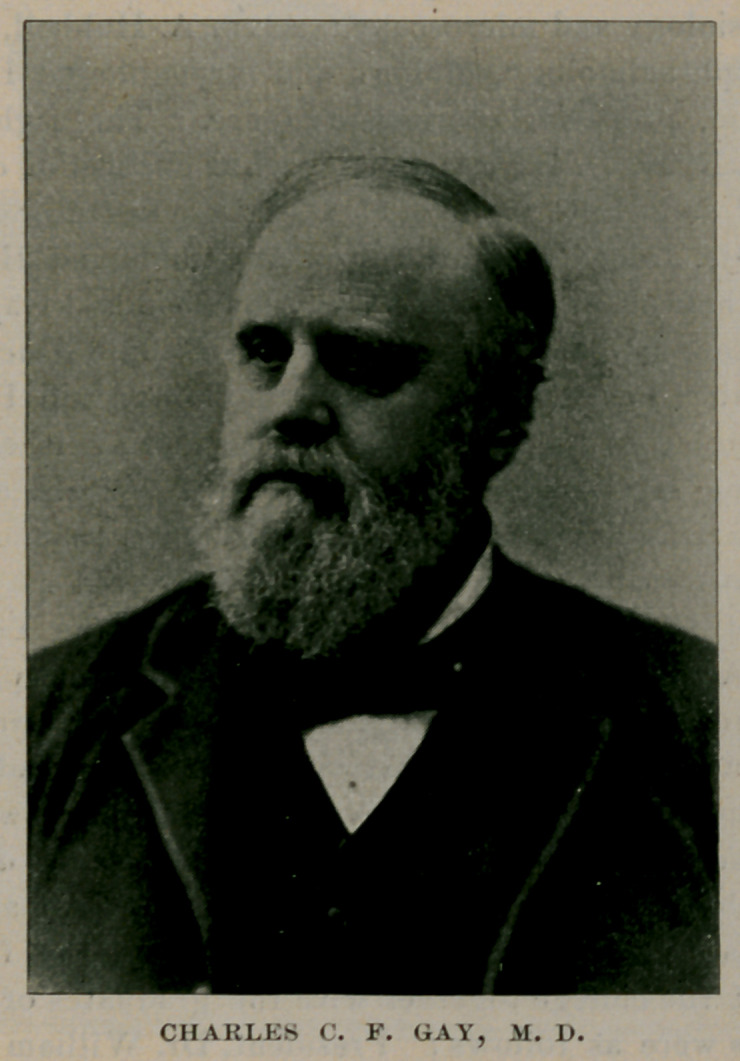


**Figure f14:**
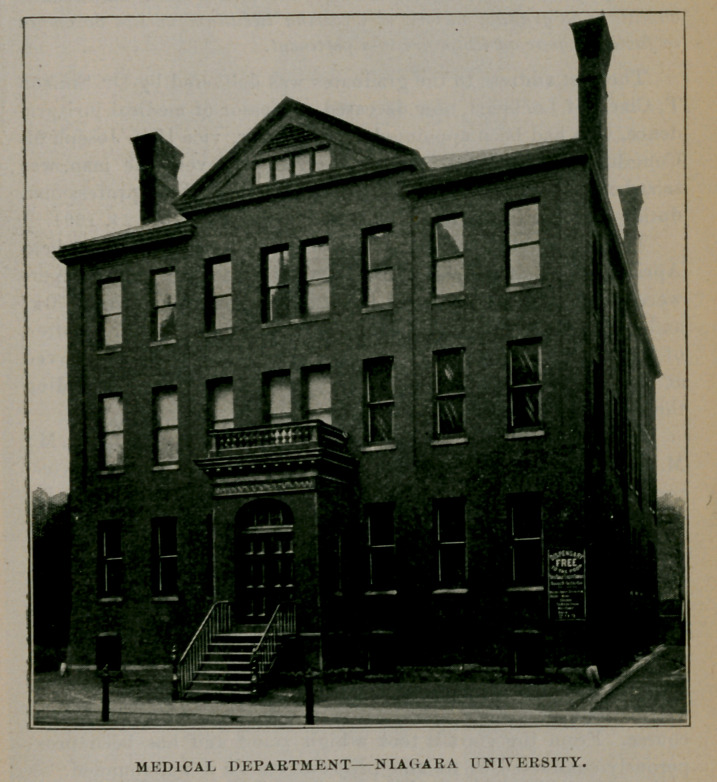


**Figure f15:**
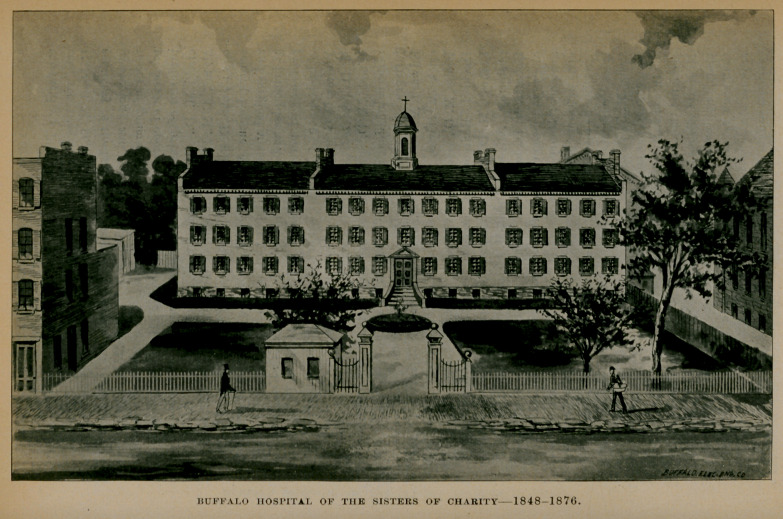


**Figure f16:**
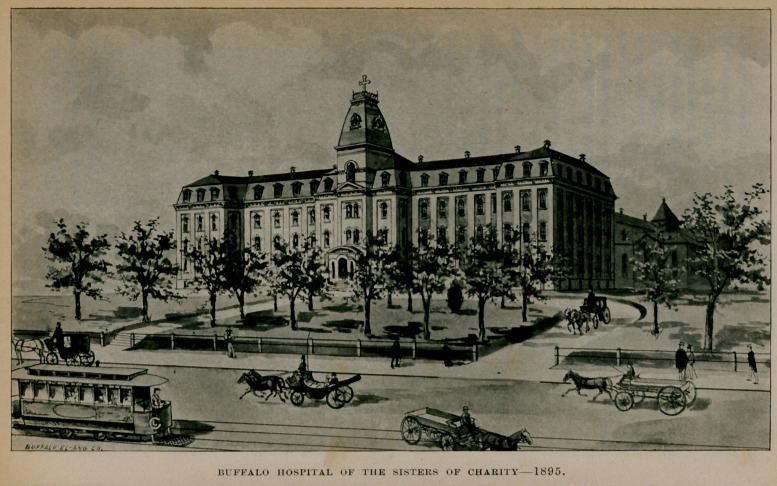


**Figure f17:**
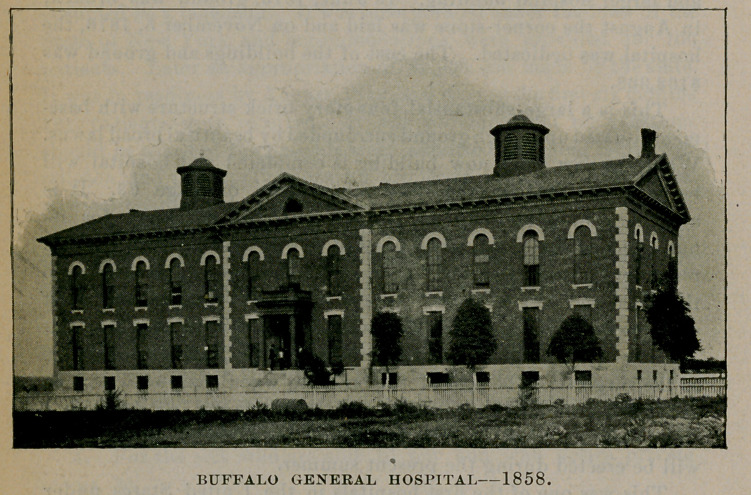


**Figure f18:**
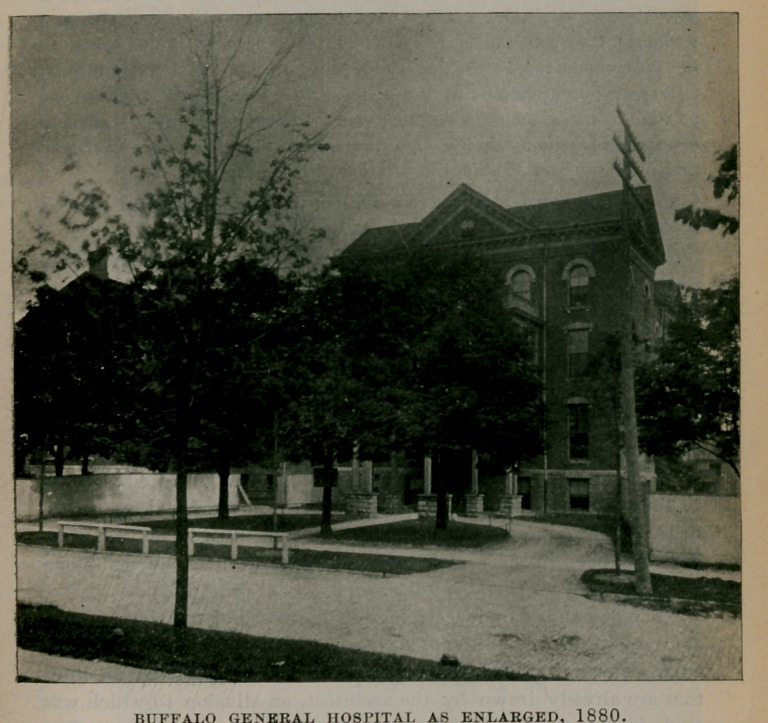


**Figure f19:**
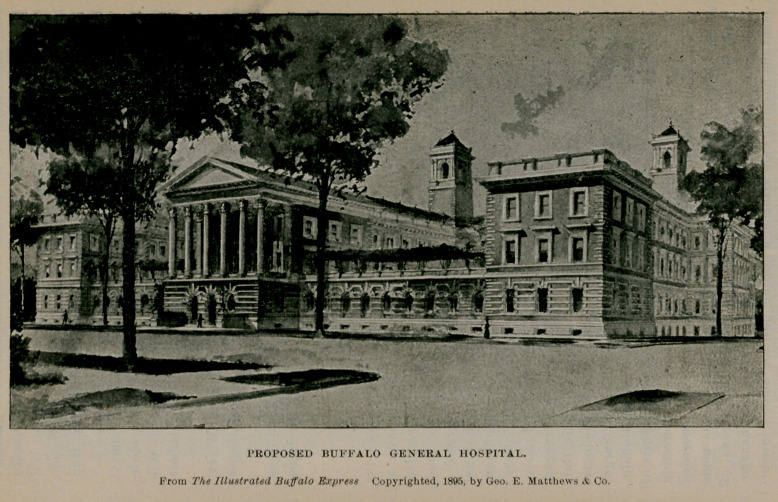


**Figure f20:**
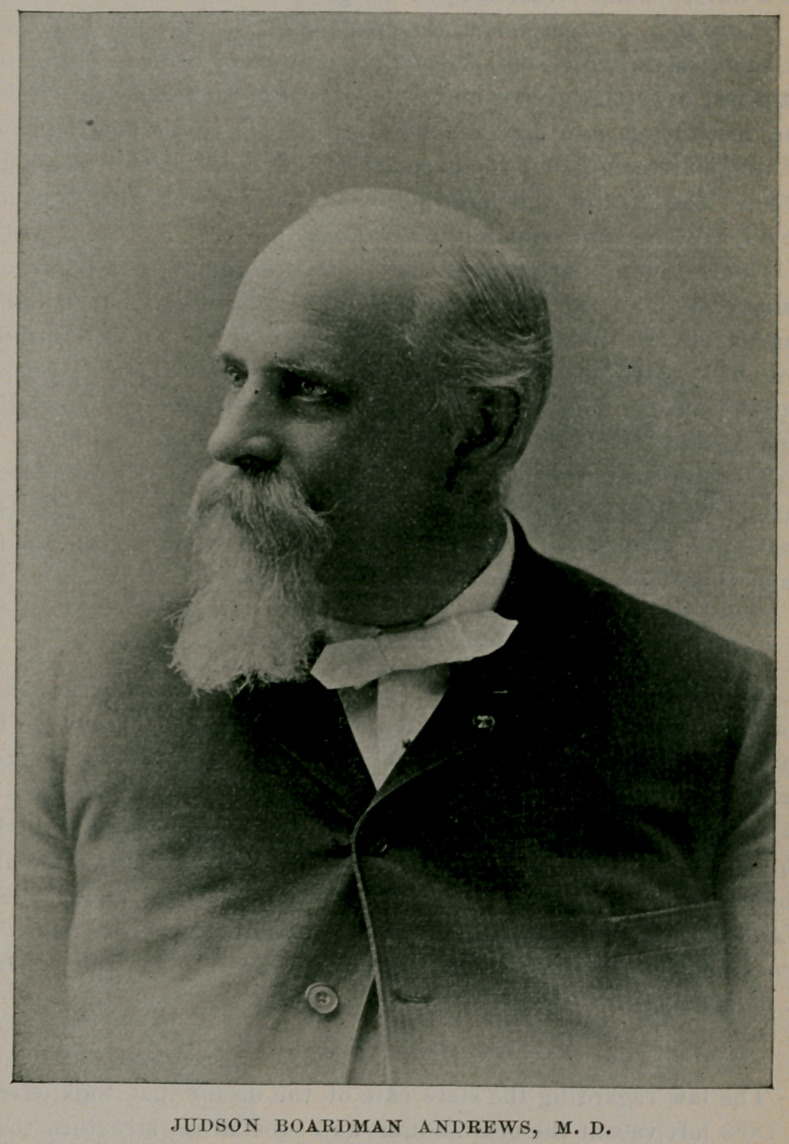


**Figure f21:**
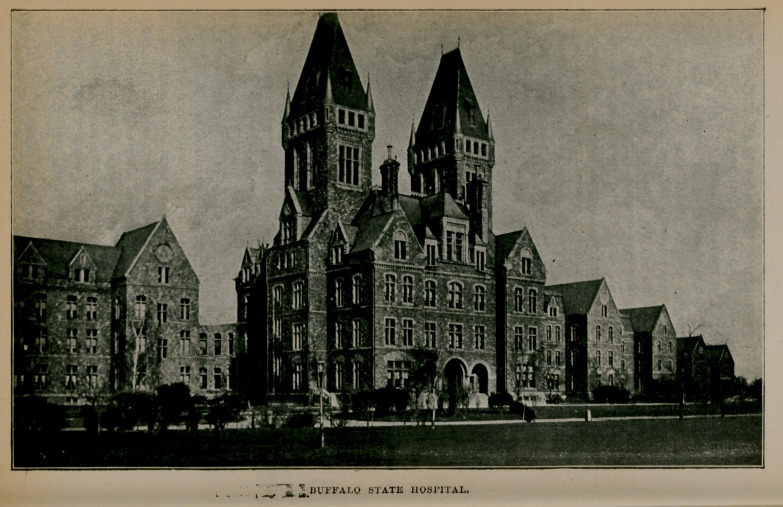


**Figure f22:**
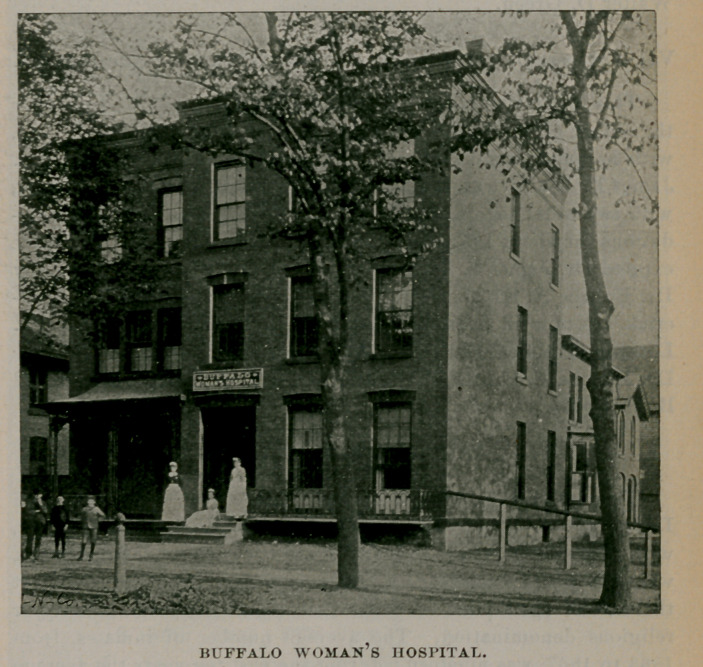


**Figure f23:**
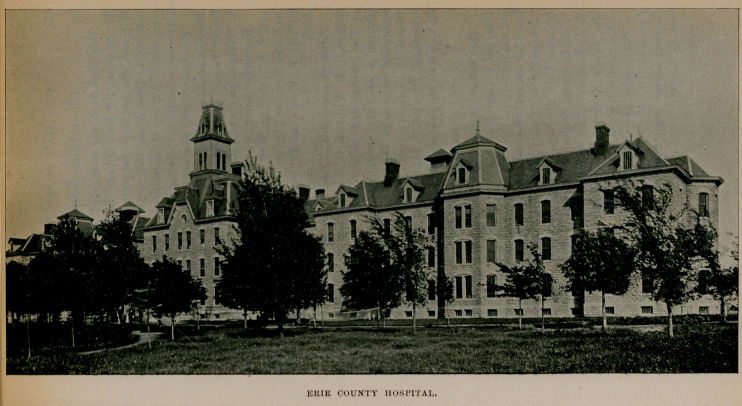


**Figure f24:**
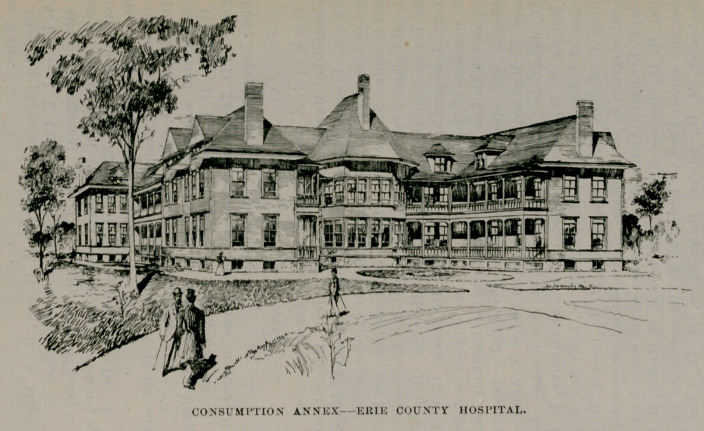


**Figure f25:**
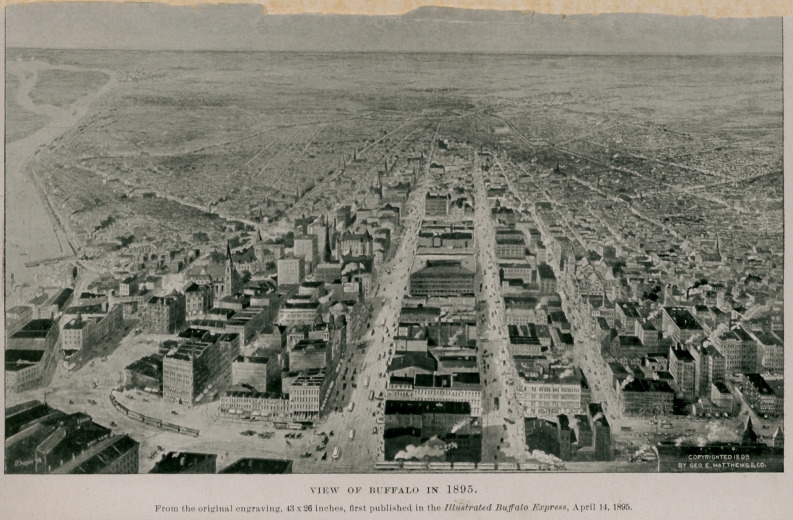


**Figure f26:**
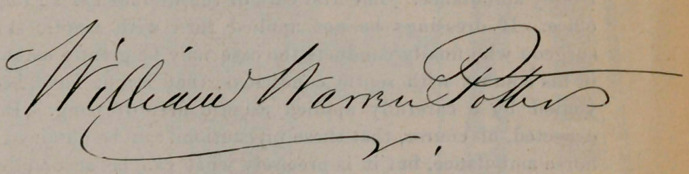


**Figure f27:**
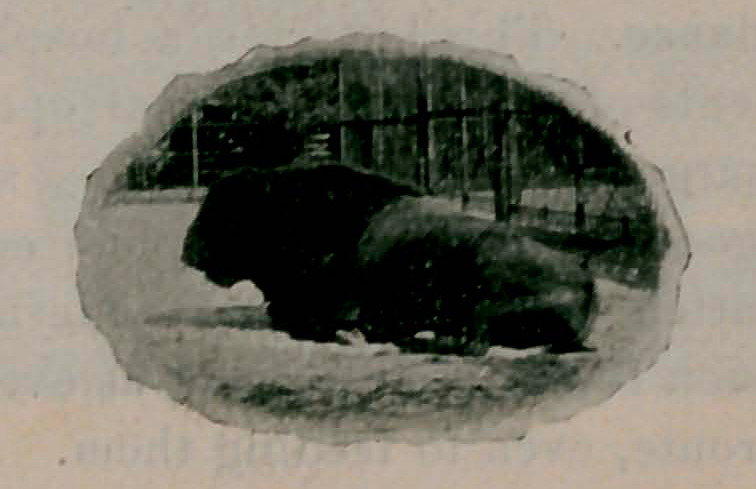


**Figure f28:**